# Immune Checkpoint Inhibitors and Immunomodulators for Cancer Immunotherapy: Insights Into Resistance and Therapeutic Strategies

**DOI:** 10.1002/advs.202521355

**Published:** 2026-04-02

**Authors:** Fangquan Chen, Yang Yu, Xiutao Cai, Junhao Lin, Ruirui Liang, Rui Kang, Daolin Tang, Jiao Liu

**Affiliations:** ^1^ DAMP Laboratory The Third Affiliated Hospital Guangzhou Medical University Guangzhou Guangdong China; ^2^ DAMP Laboratory Department of Critical Care Medicine the Third Affiliated Hospital Guangzhou Medical University Guangzhou China; ^3^ Department of Gastrointestinal Surgery The First Affiliated Hospital of Guangzhou University of Traditional Chinese Medicine Guangzhou Guangdong China; ^4^ Department of Surgery UT Southwestern Medical Center Dallas Texas USA

**Keywords:** cancer immunotherapy, cell death, immune checkpoint, immune escape

## Abstract

Cancer immunotherapy has redefined cancer treatment. However, the molecular and cellular basis of immune evasion and therapeutic resistance remains incompletely understood. Early immune checkpoint inhibitors have delivered significant clinical benefit, but their efficacy and durability remain limited in many patients. These limitations have driven the exploration of next‐generation immune checkpoints and additional regulatory pathways that shape tumor‐immune interactions. Recent advances have broadened the immune checkpoint landscape and revealed new targets. These targets operate within interconnected networks shaped by tumor‐intrinsic alterations, microenvironmental cues, the microbiome, and neuroimmune crosstalk. The application of emerging technologies has enabled high‐resolution dissection of immune‐tumor dynamics, providing a technological foundation for improving clinical outcomes through precise patient stratification and intervention. Furthermore, distinct regulated cell deaths, including apoptosis, ferroptosis, pyroptosis, necroptosis, and alkaliptosis, are increasingly recognized as critical modulators of antitumor immunity. Harnessing these mechanisms offers a rational path toward designing targeted and controllable therapeutic strategies that enhance the efficacy and durability of cancer immunotherapy.

## Graphical abstract

The schematic diagram illustrates the roles of novel immune checkpoints, immunomodulatory factors, cell death and multimodal technologies in cancer immunotherapy.

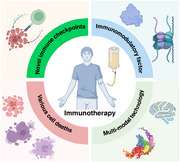

## 1 Introduction

Cancer immunotherapies have fundamentally reshaped oncology by inducing durable responses and, in some cases, complete remission in previously refractory malignancies [1]. Immune checkpoint inhibitors (ICIs), particularly antibodies targeting programmed cell death 1 (PDCD1) and cytotoxic T‐lymphocyte–associated protein 4 (CTLA4), have established immune reactivation as a clinically effective therapeutic paradigm and have produced unprecedented survival benefits across multiple cancer types [2, 3, 4].

Accumulating evidence indicates that resistance to cancer immunotherapy arises from interconnected, multilayered mechanisms rather than isolated molecular defects. Tumor‐intrinsic alterations, such as impaired antigen presentation and disrupted interferon‐γ (IFNG) signaling, limit immune recognition and responsiveness [5, 6]. Concurrently, extrinsic features of the tumor microenvironment (TME)—including immunosuppressive metabolites, aberrant stromal architecture, and dysfunctional vasculature—restrict immune cell infiltration and effector function [7]. Beyond the local niche, systemic factors such as host metabolism, the gut microbiome, and neural regulation further shape antitumor immunity and therapeutic outcomes [8, 9]. Together, these interacting layers highlight the limitations of single‐pathway targeting and emphasize the need for an integrated framework of tumor–immune regulation.

However, the conceptual scope of immune checkpoint biology has expanded beyond classical receptor–ligand signaling axes. Recent studies have identified a growing array of next‐generation checkpoints and regulatory pathways operating within complex networks that connect tumor cells [10], immune populations, stromal components, microbial communities [11], and neuroimmune circuits [12, 13, 14]. These checkpoints do not act as binary switches but function in a dynamic, context‐dependent manner, contributing to immune evasion, therapeutic resistance, and heterogeneous clinical responses.

Parallel advances in enabling technologies—such as single‐cell and spatial multi‐omics [15], high‐dimensional imaging, and systems immunology—now allow high‐resolution dissection of immune–tumor interactions in situ [16]. These approaches have revealed previously unappreciated cellular states, spatial constraints, and regulatory circuits that govern immune activation. Such insights provide a technological foundation for refining patient stratification, identifying rational combination strategies, and developing more precise, durable immunotherapies.

Within this evolving landscape, regulated forms of cell death have emerged as critical modulators of antitumor immunity. Distinct death modalities—including apoptosis, ferroptosis, pyroptosis, necroptosis, and alkaliptosis—differentially shape immune recognition, inflammatory signaling, and immune memory formation [17, 18]. Rather than serving as mere terminal endpoints, these processes actively instruct immune responses, capable of reinforcing immune evasion or enhancing immunogenicity depending on context. Thus, harnessing regulated cell death pathways represents a promising and potentially controllable strategy to overcome resistance and potentiate the efficacy of cancer immunotherapy.

In summary, tumor immunotherapy can be understood through a unified framework in which therapeutic outcome is determined by four interdependent layers: checkpoint signaling, resistance mechanisms, cell‐death‐mediated immunomodulation, and therapeutic optimization. The first layer defines the inhibitory circuits that restrain antitumor immunity; the second explains why blockade of these circuits often produces incomplete or transient responses; the third shows how regulated cell death reshapes antigen availability, inflammatory tone, and immune recruitment; and the fourth translates these insights into rational combination strategies and patient stratification. Guided by this framework, this review focuses on four themes: (1) novel immune checkpoint signaling; (2) resistance to tumor immunotherapy; (3) the immunomodulatory role of cell death; and (4) strategies to optimize immunotherapy.

## 2 The Expanding Molecular Network of Immune Checkpoints in Cancer Immunotherapy

Within the framework outlined above, the expanding checkpoint network represents the first layer of immune regulation because it defines the proximal inhibitory architecture that constrains T‐cell activation, effector function, and intercellular coordination in the tumor microenvironment. This section therefore moves from established ICIs to emerging checkpoints to show that tumor immune escape is not mediated by a single receptor‐ligand pair, but by a dynamic and interconnected suppressive system.

Immune checkpoints are essential regulators that prevent autoimmunity and maintain self‐tolerance; however, tumors frequently co‐opt these pathways to establish an immunosuppressive microenvironment and evade immune destruction [19]. Classical immune checkpoints—such as the PDCD1, CD274 (also known as PD‐L1), CTLA4, lymphocyte activating 3 (LAG3), hepatitis a virus cellular receptor 2 (HAVCR2; also known as TIM3), and v‐set immunoregulatory receptor (VSIR; also known as VISTA)—have been extensively reviewed [19, 20, 21, 22, 23]. These are canonically defined as inhibitory receptor–ligand pairs that directly transmit suppressive signals through defined immunoreceptor motifs to dampen T cell activation and effector function.

Despite the remarkable clinical success of therapeutics targeting these pathways, primary and acquired resistance remains a significant hurdle, limiting durable responses to a subset of patients (Table 1). Moreover, the efficacy of current checkpoint inhibitors is largely confined to immunologically “hot” tumors with pre‐existing T cell infiltration, leaving many “cold” tumors—such as microsatellite‐stable colorectal cancer or pancreatic ductal adenocarcinoma—largely unresponsive. These clinical challenges, coupled with the imperative to extend immunotherapeutic benefits to broader patient populations, underscore the need to explore next‐generation immune checkpoints and immunomodulatory molecules that operate beyond the classical receptor–ligand paradigm.

**TABLE 1:** The mechanism of action and challenges of classic immune checkpoint inhibitors.

Receptors	Mechanism(s) of action	Representative agents (and most advanced status)	Status	Main dilemma	Refs
PDCD1 or CD274	Expressed on activated T cells and other immune cells, it transmits inhibitory signals that weaken T cell antitumor activity	Pembrolizumab (approved), nivolumab (approved)atezolizumab (approved)	Approved	In cold tumors therapeutic efficacy is poor; primary and secondary resistance are common; some patients may experience immune‐related adverse events	[194, 195, 196]
CTLA4	Competition with CD28; inhibition of T cell activation (?)	Lpilimumab (approved)	Approved	Favorable efficacy only in certain tumors; high rate of immune‐related adverse events; controversial antitumor mechanism (e.g., Treg elimination via ADCP effects).	[197, 198, 199]
LAG3	Competition with CD4, inhibition of T cell activation (?)	Relatlimab (approved), fazelelimab (phase 3)	Limited approval	The specific signaling pathways and regulatory mechanisms are not fully understood; monotherapy is often ineffective and is often combined with PD‐1 inhibitors; there are no reliable predictive biomarkers	[200]
HAVCR2	Expressed on multiple immune cell types, it suppresses T cell function by interacting with its ligands, and is frequently co‐expressed with PDCD1 on exhausted T cells.	Sabatolimab (phase 3), cobolimab (phase 3)	Some programs terminated, others ongoing	Functional diversity may result in differing or even opposing effects under varying cellular environments and ligands; Lack of highly efficient and specific targeted therapeutics; Prone to cross‐talk with other checkpoint pathways;	[201]
VSIR	It is expressed on myeloid cells and T cells and functions as an inhibitory receptor or ligand	CA‐170 (phase 1/2)	Some programs terminated, others ongoing	The precise receptors and signaling pathways remain unclear; drug development poses challenges and requires validation; its role in immune homeostasis and tumor immunity warrants investigation	[202]

**FIGURE 1:**
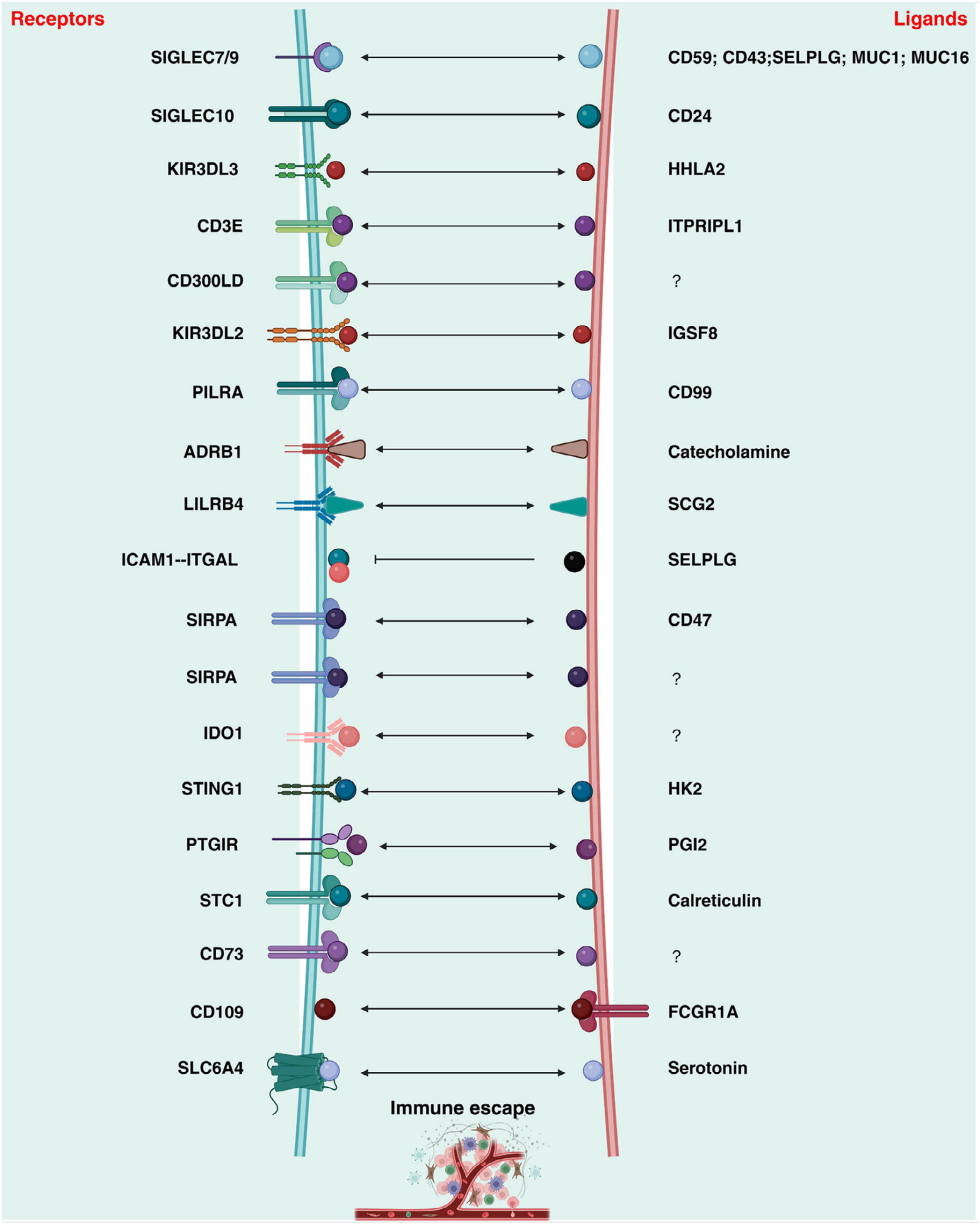
The expanding molecular network of emerging immune checkpoints in the tumor microenvironment. This schematic summarizes next‐generation inhibitory/co‐inhibitory checkpoints and metabolic checkpoint axes that collectively constrain antitumor immunity and promote immune escape in the TME. Inhibitory receptor–ligand interactions are highlighted by representative pairs linking tumor or stromal ligands to immune inhibitory receptors, including SIGLEC7/9/10 engaging sialylated ligands (e.g., CD24, CD43, CD59, SELPLG, MUC1, MUC16), KIR3DL3–HHLA2, ITPRIPL1–CD3E, IGSF8–KIR3DL2, PILRA–CD99, SCG2–LILRB4, SELPLG–ICAM1/ITGAL, and the SIRPA–CD47 axis (with SIRPA emphasized as a functional inhibitory node). These pathways converge on suppressed T‐ and NK‐cell effector signaling, T‐cell exhaustion, impaired immune synapse/activation, and reduced myeloid phagocytic or antigen‐supporting function. In parallel, the figure captures metabolic and neuro‐metabolic constraints that operate as complementary “non‐receptor” brakes on immune fitness, including IDO1‐mediated tryptophan catabolism, the STING1–HK2 metabolic checkpoint module, ADRB1–catecholamine signaling, the PGI2–PTGIR axis, STC1–calreticulin sequestration (blocking pro‐phagocytic “eat‐me” signaling), CD73‐dependent adenosine production and its upstream regulation (e.g., FCGR1A–CD109), and SLC6A4–serotonin modulation. Together, these components illustrate how inhibitory receptor–ligand checkpoints and metabolic checkpoints form an interconnected regulatory network.

Conceptually, emerging or non‐classical checkpoints encompass a broader spectrum of immunoregulatory mechanisms. These include metabolic enzymes that alter nutrient availability, lectin receptors that modulate antigen uptake, and myeloid‐associated receptors that shape the inflammatory milieu. Rather than delivering a direct inhibitory signal to T cells, these molecules indirectly sculpt immune cell fitness, signaling thresholds, and functional states within the tumor microenvironment (TME). As summarized in Figure 1 and Table 2, these emerging targets can be broadly grouped into two categories: (i) inhibitory/co‐inhibitory checkpoints that directly suppress T cell and NK cell effector function, and (ii) metabolic checkpoints that constrain anti‐tumor immunity through nutrient competition or the production of immunosuppressive metabolites.

**TABLE 2:** Some novel immune checkpoints and their mechanisms of action.

Name	Function in immunotherapy	Mechanism	Model	Ref
Siglec 7/9	Inhibition	By binding to the “anti‐phagocytic” protein (such as CD59) on prostate cancer cells, thereby inhibiting the immune activity of T cells and other immune cells	PC3, DU145, LNCaP, C42B, 22Rv1 cell lines and mice	[24]
Siglec 9	Inhibition	Inhibition of antigen presentation, secretion of chemokines, and interaction with co‐stimulatory molecules	CT2A; GL261 cell lines and mice	[25]
CD24	Inhibition	CD24 inhibits phagocytosis and promotes immune escape by interacting with Siglec 10	NCI‐H82; MCF‐7; PANC; U87‐GM cell lines and NSG mice	[26]
KIR3DL3	Inhibition	KIR3DL3 binds HHLA2 to inhibit TCR signaling	K562 and Jurkat cell lines	[27]
ITPRIPL1	Inhibition	The binding of ITPRIPL1 extracellular domain to CD3E on T cells significantly decreased calcium influx and ZAP70 phosphorylation, impeding initial T cell activation.	HCT116; SW1116; SW48; MDA‐MB‐231; Jurkat; Raji; RL; HL‐60; H446; RD; A549; H1299; 143B; KHOS; B16; MC38; CHO; P815 cell; mice	[28]
CD300LD	Inhibition	STAT3 regulates the expression of S100A8/9, thereby mediating the migration and recruitment of PMN‐MDSCs and suppressing T cell activation	B16‐F10; MC38; LLC; EL4; mice	[29]
IGSF8	Inhibition	IGSF8 expressed on tumors suppresses NK cell function by interacting with human KIR3DL2	K562; PC9; LLC; COLO205; CT26; A375; B16‐F10; EMT6 cell lines; mice	[10]
PILRA	Inhibition	PILRA bind to CD99 to inhibit T cell activation and effector function by suppressing ZAP70/NFAT/IL‐2/JAK/STAT signaling	Hepa1‐6; MDA‐MB‐231; U87; mice	[30]
ADRB1	Inhibition	Catecholamine promotes T cell exhaustion and inhibits cytokine secretion through ADRB1	YUMMER1.7; MC38; 6419c5; mice	[31]
SCG2	Inhibition	SCG2 binds to LILRB4 receptor on monocyte surfaces, activating the IL6‐STAT3 pathway to reprogram myeloid cell function	A375; B16F10; THP‐1; MC38; EO771; CT2A; 2B4 cell lines; mice	[32]
SELPLG	Inhibition	SELPLG can promote T cell exhaustion and by blocking interactions between tumor cell ICAM1 and macrophage ITGAL	YUMMER1.7; B16‐OVA; OT‐I T; B16‐GP33; L1210; EL4; L929; ARP‐1; Molm‐13; KG1a; MM1.S; D4M‐3A; mice	[33, 34, 35]
SIRPA	Inhibition	The interaction of SIRPA with CD47 inhibits the phagocytosis of macrophages	Various leukemia cell lines and mice	[36, 37, 38]
IDO1	Inhibition	Catalyzes tryptophan metabolism to produce metabolites such as kynurenine, thereby promoting regulatory T cell differentiation and suppressing immune responses	Various tumors and mice	[44, 45, 46]
STING1	Promotion	STING1 targets HK2 to block its hexokinase activity, thereby restricting tumor aerobic glycolysis and promoting anti‐tumor immunity	U2OS; HeLa; HCT116; LoVo; RKO; MC38; CT26; L929 cell lines and mice	[47]
PTGIR	Inhibition	PTGIR signaling impairs T cell metabolism and cytokine production via NFE2L2 pathway	B16‐OVA; MC38‐OVA cell lines; mice	[48]
STC1	Inhibition	Traps calretinolin within mitochondria, blocking the eat‐me signal and inhibiting phagocytic function in macrophages and dendritic cells	Melanoma and colorectal cancer cell lines and mice	[49]
CD73	Inhibition	Inhibits immune cell function by catalyzing the conversion of AMP to adenosine.	MDA‐MB‐231; A375; BxPC‐3; HepG2; MC38 cell lines; mice	[52, 53, 55]
CD109	Inhibition	sCD109 upregulates *CD73* mRNA transcription by activating the FCGR1A/SYK/NF‐κB signaling pathway	RBE; HCCC‐9810; LTP‐C9; PANC04.03; KP‐2; KPC; LN229; U251; J774a.1 cel; THP‐1 cell lines and mice	[54]
SLC6A4	Inhibition	Suppresses the 5‐HTR‐ MAPK pathway by clearing serotonin, thereby weakening the antitumor activity of CD8^+^ T cells	B16‐OVA; B16‐F10; A375; MC38; CT26; PG13 cell; MB49; 4T1; PC3 cell lines and mice	[56]
CLEC12B	Inhibition	Inhibition of NK cell activity	HCC, colorectal cancer and melanoma mouse model	[203]

Understanding these extended regulatory layers is critical, as it helps explain why blocking classical receptor–ligand interactions alone is often insufficient in “cold” tumors or in acquired resistance settings. In the following sections, we first outline the key inhibitory/co‐inhibitory checkpoints, then expand to discuss metabolic checkpoints, and finally connect these pathways to non‐checkpoint immunomodulators, resistance mechanisms, and emerging therapeutic strategies.

### 2.1 Inhibitory and Co‐Inhibitory Checkpoints

Inhibitory and co‐inhibitory checkpoints, as introduced above, serve as critical signaling hubs that translate sustained immunosuppressive cues from the tumor microenvironment (TME) into functional exhaustion of cytotoxic lymphocytes. This persistent checkpoint engagement thereby establishes a mechanistic axis directly connecting TME‐mediated suppression to clinical outcomes such as non‐response and relapse.

Sialic acid binding Ig like lectins (SIGLECs), expressed on tumor‐associated macrophages (TAM), suppress T‐cell and innate immune activity by engaging “don't‐eat‐me” signals such as CD59 on tumor cell surfaces, thereby promoting immune escape in prostate, ovarian, triple‐negative breast, and glioblastoma cancers [24, 25, 26]. In NSCLS and metastatic melanoma, killer cell immunoglobulin like receptor, three Ig domains and long cytoplasmic tail 3 (KIR3DL3)‐highly expressed on NK and T cells‐binds to HHLA2 member of B7 family (HHLA2) on tumor cells, activating the immune receptor tyrosine inhibitory motif (ITIM) pathway. This pathway recruits tyrosine phosphatases protein tyrosine phosphatase non‐receptor type 6 (PTPN6; also known as SHP1) and PTPN11 (SHP2), ultimately suppressing NK cell cytotoxicity and T cell activation [27]. Similarly, ITPRIP like 1 (ITPRIPL1; also known as CD3‐L1) directly binds to CD3 epsilon subunit of T‐cell receptor complex (CD3E) on the T cells, inhibiting calcium influx and phosphorylation of zeta chain of T cell receptor associated protein kinase 70 (ZAP70). This impairs downstream T‐cell signaling and weakens antitumor activity in models of colon cancer and melanoma [28]. CD300 molecule like family member D (CD300LD) is highly expressed in polymorphonuclear myeloid‐derived suppressor cells (PMN‐MDSCs), where it promotes immunosuppression and tumor escape via the signal transducer and activator of transcription 3 (STAT3)‐S100 calcium binding protein A8 (S100A8)/A9 axis, facilitating tumor escape in multiple mouse models [29].

Other inhibitory pathways further diversify immune evasion mechanisms. Immunoglobulin superfamily member 8 (IGSF8), a tumor‐cell transmembrane protein, engages the NK‐cell receptor killer cell immunoglobulin like receptor, three Ig domains and long cytoplasmic tail 2 (KIR3DL2) to inhibit NK‐cell cytotoxicity in B16‐F10 melanoma, CT26 colon cancer, and EMT6 breast cancer models [10]. Similarly, paired immunoglobin like type 2 receptor alpha (PILRA) suppresses T‐cell activation by binding CD99 and attenuating the ZAP70/nuclear factor of activated T cells (NFATs)‐interleukin 2 (IL2)‐janus kinase (JAK)‐STAT signaling pathway, thereby promoting immune escape in mouse models of glioma and triple‐negative breast cancer [30].

Beyond canonical immune checkpoints, neuroimmune and metabolic pathways also exert co‐inhibitory control. Activation of β‐adrenergic receptor 1 (ADRB1) signaling by sympathetic neurotransmitter norepinephrine induces CD8^+^ T‐cell exhaustion and enhances tumor growth in pancreatic, colon, and melanoma models [31]. Secretogranin II (SCG2) binds to the leukocyte immunoglobulin like receptor B4 (LILRB4) receptor on monocytes, activating the IL6‐STAT3–dependent myeloid reprogramming and tumor progression [32]. Selectin P ligand (SELPLG) promotes T cell exhaustion [33, 34], and its disruption—by blocking tumor‐cell intercellular adhesion molecule 1 (ICAM1) interaction with macrophage integrin subunit alpha L (ITGAL) [35]‐restores antitumor responses in melanoma and hematologic malignancies.

The signal regulatory protein α (SIRPA)–CD47 axis represents another well‐characterized inhibitory pathway. Engagement of CD47 on tumor cells with SIRPA on macrophages delivers a “don't‐eat‐me” signal that blocks phagocytosis and supports immune evasion, particularly in hematologic malignancies with high CD47 expression [36, 37, 38]. However, clinical outcomes targeting the SIRPA‐CD47 axis often yield suboptimal benefits. This may be because a significant portion of SIRPA's inhibitory function is independent of CD47 and instead achieved through cis‐interactions with CD18 on the macrophage surface [39]. Additional evidence suggests that SIRPA can autonomously suppress antitumor immunity in colorectal, lung, and liver cancers, indicating that targeting SIRPA directly may represent a more effective strategy than CD47 blockade alone [40].

Collectively, these findings reveal that tumor immune evasion extends well beyond classical PD‐1/PD‐L1 signaling. While combining novel checkpoint inhibitors with existing immunotherapies or conventional therapies offers a promising strategy to overcome resistance, a deeper layer of regulation persists. Even when receptor–ligand interactions are blocked, immune function can be suppressed by non‐receptor mechanisms—most notably metabolic competition and immunosuppressive metabolites. This highlights metabolic checkpoints as a critical, complementary axis of immune regulation and therapeutic resistance.

### 2.2 Metabolic Checkpoints

Metabolic checkpoints represent a fundamental layer of immune control, arising from the metabolic competition between tumor cells and immune cells within the nutrient‐deprived tumor microenvironment [41]. By integrating metabolic cues with immune signaling, these pathways critically shape the fate and function of infiltrating lymphocytes and myeloid cells, ultimately determining whether antitumor immunity is sustained or suppressed.

Indoleamine 2,3‐dioxygenase 1 (IDO1) primarily catalyzes tryptophan metabolism to produce metabolites such as kynurenine, thereby promoting regulatory T cell differentiation and suppressing immune responses across various tumors [42, 43]. The clinical efficacy of the IDO1 inhibitor epacadostat, in combination with the PDCD1 inhibitor pembrolizumab, appears to be tumor type–dependent [44, 45]. Novel IDO1 inhibitors, including BMS‐986205, are currently under clinical investigation in combination with ICIs for advanced solid and hematologic malignancies [46].

The stimulator of interferon response CGAMP interactor 1 (STING1) also exerts metabolic control by binding and inhibiting hexokinase 2 (HK2), thereby reducing lactate production, enhancing CD8^+^ T‐cell infiltration, and augmenting antitumor immunity in colorectal cancer models [47]. This interaction represents a noncanonical checkpoint mechanism distinct from classical receptor–ligand interactions. Similarly, the oxidative stress regulator NFE2 like BZIP transcription factor 2 (NFE2L2, also known as NRF2) exerts dual, context‐dependent effects on CD8^+^ T‐cell function: while mitigating oxidative damage through glutathione synthesis, it also induces prostaglandin I_2_ receptor (PTGIR) expression, driving T‐cell exhaustion and immune escape in melanoma and colon cancer models [48].

Stanniocalcin 1 (STC1) has been identified as an additional metabolic checkpoint that sequesters calretinolin within mitochondria, blocking the eat‐me signal and inhibiting phagocytic function in macrophages and dendritic cells. This mechanism correlates with immunotherapy resistance in several solid tumors, including melanoma and colorectal cancer [49, 50].

Adenosine metabolism constitutes another major axis of metabolic immunosuppression. CD73, the rate‐limiting enzyme in the extracellular purine metabolism pathway, catalyzes the conversion of AMP to adenosine, dampening immune activation. CD73 inhibitors such as oleclumab are currently under clinical evaluation in multiple malignancies [51, 52, 53]. In intrahepatic cholangiocarcinoma, CD109 enhances CD73 stability through the Fc Gamma Receptor Ia (FCGR1A)‐spleen associated tyrosine kinase (SYK)‐nuclear factor kappa b subunit (NFKB) signaling cascade, suggesting that targeting CD109 may provide an upstream strategy to suppress adenosine‐mediated immunosuppression [54]. However, loss of CD73 function has been linked to increased thrombotic risk, underscoring the need to assess thrombogenic potential when developing CD73‐targeted therapies [55].

Serotonin metabolism also intersects with immune regulation. The serotonin transporter solute carrier family 6 member 4 (SLC6A4) reduces extracellular serotonin availability, thereby dampening 5‐hydroxytryptamine receptor (5‐HTR)‐ mitogen‐activated protein kinase (MAPK) signaling and diminishing CD8^+^ T‐cell cytotoxicity. Pharmacological inhibition of SLC6A4 with clinically used selective serotonin reuptake inhibitors (SSRIs), including fluoxetine and citalopram, restores T‐cell infiltration and antitumor activity in melanoma and colon cancer models [56].

In summary, metabolic checkpoints represent a multilayered regulatory network that integrates adaptive and innate immunity, metabolic adaptation, and microenvironmental cues. Understanding how these pathways intersect with cytokine networks, stromal dynamics, and chromatin remodeling will be essential for overcoming metabolic resistance and broadening the responsiveness of immunologically “cold” tumors. As these checkpoints are deeply embedded within the broader tumor–immune ecosystem, their therapeutic targeting must be considered within this integrated framework.

## 3 Molecular Mechanisms of Immunomodulators in Anti‐Tumor Immunity

**FIGURE 2:**
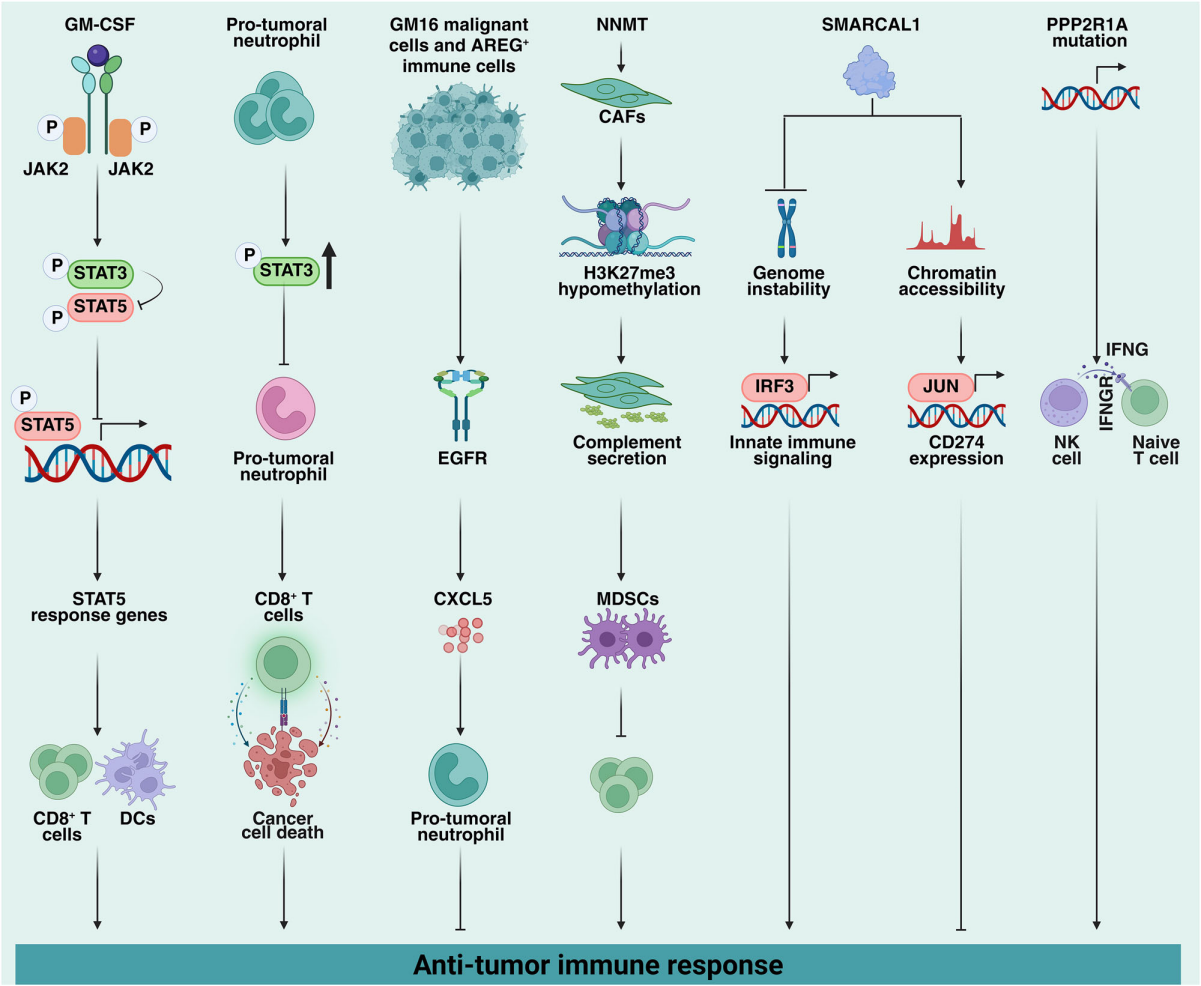
The molecular mechanisms of immunomodulators in antitumor immunity. Immunoregulatory molecules exert dual roles in antitumor immunity through complex network interactions to reshape the tumor microenvironment. For instance, excessive STAT3 activation impairs dendritic cell function by inhibiting JAK2‐STAT5 signaling mediated by granulocyte‐macrophage colony‐stimulating factor (GM‐CSF). Moreover, abnormal STAT3 activation in pro‐tumor neutrophils can drive tumor‐associated neutrophils toward an immunosuppressive phenotype, inhibiting CD8^+^ T cell killing activity. This duality of regulation is similarly reflected in the EGFR signaling network—AREG upregulates CXCL5 expression by activating EGFR, recruiting immunosuppressive neutrophils to promote metastasis. At the epigenetic level, NNMT induces H3K27me3 demethylation in CAFs to trigger complement factor secretion, thereby recruiting MDSCs to form an immunosuppressive microenvironment. Meanwhile, SMARCAL1 mediates immune evasion through dual mechanisms: suppressing the CGAS‐STING1 pathway to reduce tumor immunogenicity while maintaining *CD274* gene accessibility to promote PD‐L1 expression. Additionally, *PPP2R1A* mutations transform cold tumors into hot tumors by enhancing IFNG signaling and recruiting immune cells.

Beyond the checkpoint axis itself, a growing repertoire of immunomodulatory factors governs the tumor immune microenvironment through mechanisms that extend beyond direct receptor–ligand inhibition (Figure 2 and Table 3). These regulators act as network‐level rheostats, tuning antigen presentation, myeloid polarization, stromal permeability, and exhaustion kinetics. In doing so, they set the baseline immune tone that ultimately determines whether checkpoint blockade—classical or next‐generation—can elicit durable therapeutic responses.

**TABLE 3:** Some novel immunomodulatory molecules and their mechanisms of action.

Name	Function in immunotherapy	Mechanism	Model	Ref
STAT3	Inhibition	Inhibits the JAK2‐STAT5 pathway, impairing DC function	JAWSII; B16F10; CT26; EMT6; 4T1; LLC cell lines and mice	[57]
STAT3	Inhibition	Excessive activation of the STAT3 signaling pathway in tumor‐promoting neutrophils impair CD8^+^ T cell antitumor activity	Mice	[58]
AREG	Inhibition	Upregulates CXCL5 expression via EGFR signaling, recruiting neutrophils	GBC‐SD; SGC996 cell lines and mice	[59]
NNMT	Inhibition	NNMT‐induced H3K27me3 hypomethylation drives complement secretion from CAFs, attracting immunosuppressive MDSCs to the tumor	BPPNM; HGS‐2; OVCAR8; ID8; ID8‐F3; HTB‐75; E0771‐LMB; MC38; K562 cell lines; mice	[60]
SMARCAL1	Inhibition	It suppresses the CGAS‐STING1 pathway to reduce tumor immunogenicity while cooperating with Jun to maintain *CD274* gene site accessibility, thereby enhancing its expression	T‐47D; MDA‐MB‐436; BT‐474; SK‐BR‐3; MDA‐MB‐361; BT‐20; MDA‐MB‐231; MCF7; U2OS; PC‐3; B16/F10; Renca; MC38 cell lines and mice	[61]
Mutant *PPP2R1A*	Promotion	*PPP2R1A* mutations enhance IFNG signaling, recruiting immune cells, and promote tertiary lymphoid structure formation	Recurrent, platinum‐resistant or refractory OCCC patient	[62]
CD61, CD103	Promotion	Unexpected and transient co‐expression of CD61 and CD103 in T cell synaptic microaggregates promotes TCR signaling and antigen‐specific T cell cytotoxicity.	Mice	[63]

Signal transducer and activator of transcription (STAT) family members exemplify this dynamic regulation. STAT3 and STAT5 coordinate dendritic cell (DC) differentiation and function through the JAK‐STAT pathway. In the TME, hyperactivation of STAT3 inhibits the JAK2‐STAT5 pathway, leading to impaired DC maturation and antigen presentation. Pharmacological degradation of STAT3 using small‐molecule degraders such as SD‐36 reverses this suppression, restores DC function, and enhances antitumor immunity in MC38 colon carcinoma, B16‐F10 melanoma, and 4T1 breast carcinoma models [57]. Similarly, excessive activation of the STAT3 signaling pathway in tumor‐promoting neutrophils contributes to an immunosuppressive milieu. Inhibition of STAT3 by agents such as LLL12 reprograms these neutrophils toward a proinflammatory phenotype, converting “cold” tumors into “hot” immune microenvironments and amplifying CD8^+^ T‐cell responses in mouse models of oropharyngeal carcinoma (MOPC) and melanoma (B16‐F10) [58].

Epidermal growth factor receptor (EGFR) signaling also contributes to immunoregulation. Amphiregulin (AREG) upregulates C‐X‐C motif chemokine ligand 5 (CXCL5) expression through EGFR activation, promoting neutrophil recruitment, metastasis, and immune evasion. Combined inhibition of EGFR and immune checkpoints markedly improves therapeutic efficacy and mitigates immunotherapy resistance in gallbladder cancer [59].

Epigenetic and stromal modulators likewise play pivotal roles. Nicotinamide N‐methyltransferase (NNMT) activates cancer‐associated fibroblasts (CAFs) through H3K27me3 hypomethylation, inducing complement factor secretion that recruits myeloid‐derived suppressor cells (MDSCs) and suppresses CD8^+^ T‐cell activity. NNMT inhibition reduces tumor burden and metastasis and synergizes with checkpoint blockade in ovarian, breast, and colorectal cancer models [60].

Chromatin remodeling enzyme SNF2 related chromatin remodeling annealing helicase 1 (SMARCAL1) promotes immune evasion through a dual mechanism: suppression of the cyclic GMP–AMP synthase (CGAS)–STING1 pathway to reduce tumor immunogenicity, and maintenance of *CD274* (PD‐L1) gene accessibility through cooperation with JUN. Combined SMARCAL1 deficiency and PD‐L1/CTLA4 blockade significantly inhibit melanoma growth [61]. Conversely, protein phosphatase 2 scaffold subunit alpha (*PPP2R1A*) mutations transform cold tumors like ovarian clear cell carcinoma into hot tumors by enhancing IFNG signaling, recruiting immune cells, and promoting tertiary lymphoid structure formation [62].

Additional immunomodulators have been implicated in fine‐tuning T‐cell activity. CD61 (integrin β3) transiently cooperates with CD103 to modulate T‐cell receptor (TCR) signaling, sustaining antitumor effector function while preventing overstimulation and exhaustion [63].

In summary, immune checkpoints function as critical gatekeepers that directly restrain immune activation, whereas immunomodulators orchestrate broader regulatory networks—integrating cytokine signaling, metabolic reprogramming, chromatin dynamics, and stromal crosstalk. Deciphering the mechanistic interplay between these layers will be essential for designing rational combination strategies that overcome therapeutic resistance and achieve durable clinical benefit across diverse tumor types. In the following section, we elucidate how failures at each of these interconnected levels drive resistance to immune checkpoint blockade and how mechanism‐matched interventions can restore effective antitumor immunity.

## 4 The Mechanism of Immunotherapy Resistance and Therapeutic Strategy

The second layer of the framework is resistance, which determines whether interruption of checkpoint signaling can be translated into durable tumor control. Resistance arises when tumor‐intrinsic alterations, suppressive stromal and immune niches, systemic modulators, or therapy‐driven adaptive changes prevent effective priming, infiltration, recognition, or killing despite ICI treatment. This section therefore organizes resistance mechanisms according to where the bottleneck occurs and how each bottleneck constrains the therapeutic value of checkpoint blockade.

**FIGURE 3:**
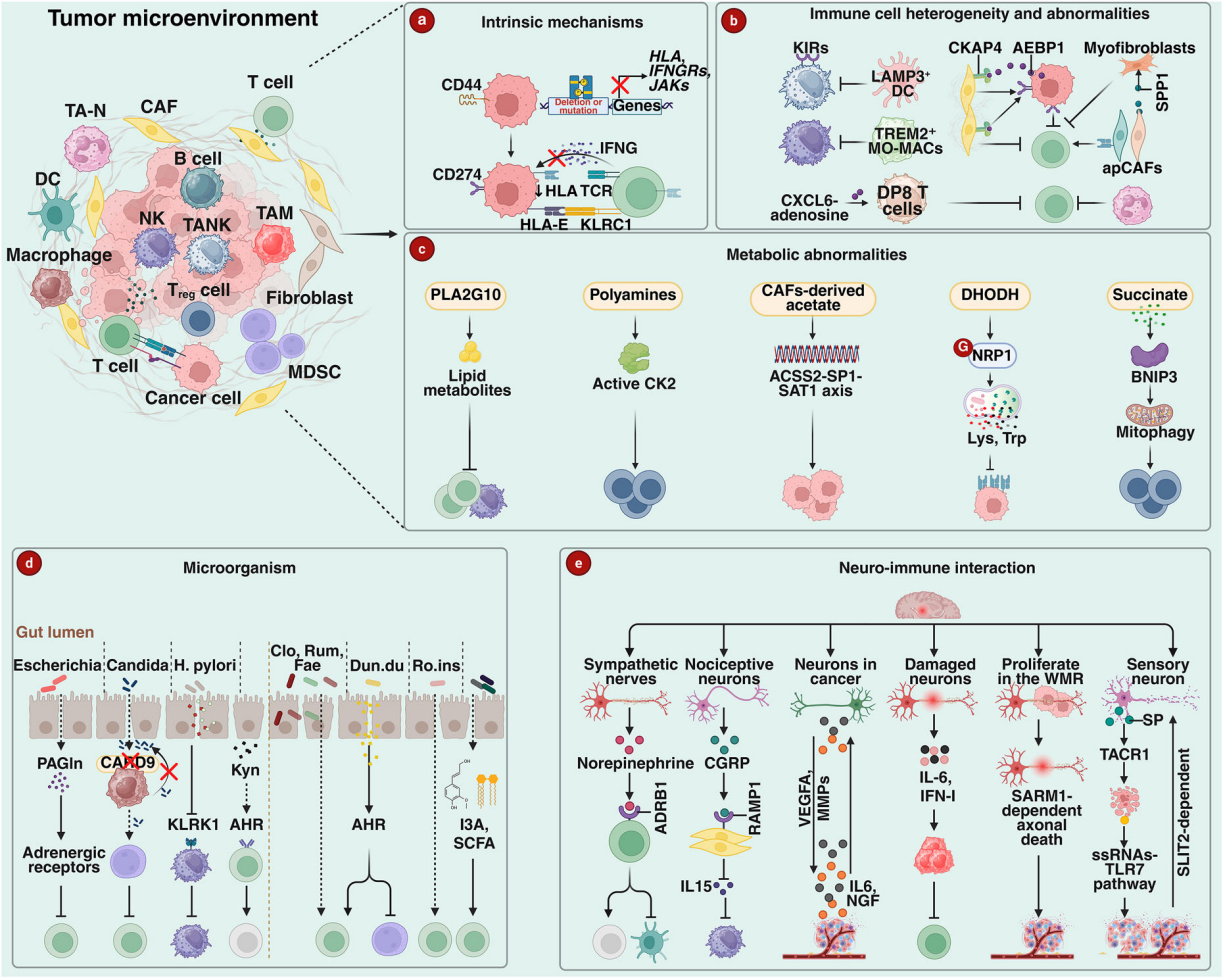
The mechanism of immunotherapy resistance. (a) Heterozygous deletions or mutations in tumor cell *HLA*, *IFNGRs*, or *JAK*s genes can lead to loss of HLA class molecules from the cell membrane, resulting in loss of neoantigen expression and promoting immune escape. Conversely, the interaction between HLA‐E and KLRG1 contributes to an immunosuppressive microenvironment. (b) Different immune cell populations in the tumor microenvironment exert opposing effects to jointly regulate immune responses. Notably, NK cell subsets exhibit significant heterogeneity in tumor tissue distribution. For instance, c6‐DNAJB1 NK (TANK) cells highly express KIRs, exhibit enhanced stress responses, and reduced cytotoxicity, while cellular interactions with LAMP3^+^ DCs shape tumor‐infiltrating CD56^dim^ CD16^hi^ NK cell functions. Furthermore, TREM2^+^ monocyte‐derived antigen‐presenting cells (MO‐MACs) within the tumor also suppress NK cytotoxicity. CAF subpopulations exhibit dual regulatory effects: tumor‐secreted AEBP1 binds to surface CKAP4 on CAFs, inducing T cell exhaustion, whereas antigen‐presenting CAFs (apCAFs) promote T cell activation through high MHCII expression. Furthermore, interactions between immune senescence and tumor cells further impact immunotherapy efficacy. For example, CXCR6‐adenosine axis‐mediated DP8 T cell activation suppresses antitumor T cell activity. (c) Tumor cells adapt to microenvironmental stress through metabolic reprogramming, while significantly impairing immune cell function within the tumor microenvironment. For instance, tumor cells promote phospholipid hydrolysis by upregulating PLA2G10, generating inhibitory lipid metabolites that compromise T cell and NK cell infiltration. Additionally, tumor‐released polyamines promote Treg cell differentiation toward an immunosuppressive phenotype via the CK2 signaling pathway. Pancreatic CAFs enhance tumor growth by secreting acetate through the ACSS2‐SP1‐SAT1 signaling axis. Tumor cells may also evade immunity by DHODH‐mediated macropinocytosis of lysine and tryptophan, which subsequently suppresses HLA class I molecule transcription. Conversely, succinate enhances CD8^+^ T cell adaptability via BNIP3‐mediated mitochondrial autophagy, thereby improving T cell survival. (d) The gut microbiota exhibits bidirectional regulation with antitumor immunity. *Escherichia* enriched in the intestines of non‐responders activates adrenergic receptors via the metabolite phenylacetylglutamine (PAGln), thereby suppressing CD8^+^ T cell function. while *Card9*‐deficient mice exhibit excessive proliferation of *Candida tropicalis*, inducing granulocyte‐like myeloid‐derived suppressor cells (G‐MDSCs). *Helicobacter pylori* suppress NK cell activity by downregulating the KLRK1 receptor. Additionally, chronic stress induces oral microbiota dysbiosis, which mediates CD8^+^ T cell exhaustion via the tyrosine‐aromatic hydrocarbon receptor (AhR) pathway. Conversely, *Clostridiales*, *Ruminococcaceae*, and *Faecalibacterium* enhance CD8^+^ T cell effector function; supplementation with *Duncaniella dubosii* reverses immunosuppression by activating the AhR pathway. *Roseburia intestinalis* produces butyrate to activate CD8^+^ T cells. Furthermore, indole‐3‐propionic acid and the short‐chain fatty acid butyrate, derived from certain gut bacteria, enhance CD8^+^ T cell effector functions. (e) The nervous system also regulates tumor immune responses. For instance, norepinephrine released by sympathetic nerves inhibits dendritic cell function and induces CD8^+^ T cell exhaustion via ADRB1 receptors, while CGRP secreted by nociceptive neurons weakens NK cell antitumor activity by suppressing IL15 production from CAFs. Furthermore, tumor‐derived IL6 and NGF reprogram neurons to express VEGFA and MMPs, driving angiogenesis and immune suppression. IL‐6 and IFN‐I released from damaged neurons recruit M2 macrophages and exhaust CD8^+^ T cells. SARM1‐mediated axonal death promotes glioblastoma progression via neuroinflammation. In breast cancer, the neuropeptide substance P activates the ssRNA‐TLR7 pro‐tumorigenic pathway via the TACR1 receptor, thereby promoting metastasis.

Resistance to immune checkpoint blockade (ICB) emerges from the complex interplay of tumor‐intrinsic adaptations, microenvironmental remodeling, and systemic immune dysregulation (Figure 3). Overcoming this multifaceted resistance requires rational strategies that reprogram the tumor microenvironment (TME), restore immune effector function, and enhance antigen presentation. As summarized in Table 4, these therapeutic approaches can be conceptually mapped to three interconnected layers of resistance: (i) tumor‐intrinsic loss of immune recognition, (ii) TME‐level barriers—including cellular/stromal constraints and metabolic suppression—and (iii) systemic host factors that shape global immune tone beyond the tumor. This layered framework underscores a key principle: improving ICB outcomes often requires matching interventions to the dominant “failing layer” rather than simply intensifying checkpoint blockade alone.

**TABLE 4:** Innovative strategies for overcoming immunotherapy resistance.

Targets or regimen	Mechanism	Model	Ref
HA and PTK2	PEGPH20‐mediated HA degradation and PTK2 inhibitors to reduce the expression of phosphorylated PTK2 synergistically enhances the therapeutic efficacy of anti‐PDCD1 antibodies	PDAC cell lines and mice	[80]
RHOT2	Targeting RHOT2 blocks cancer cells transfer mitochondria to fibroblasts, inhibiting CAF formation and tumor growth	Skin, breast, and pancreatic cancer cell lines and mice	[204]
HCAR1	Inhibiting theHCAR1 blocks the YWHAZ‐STAT3 pathway, reduce CCL2/7 secretion and CCR2^+^ PMN‐MDSC recruitment	Colorectal cancer cell lines and mice	[7]
MLXIP	Genetic or pharmacological (SBI‐477) inhibition of MLXIP–TXNIP signaling axis enhances CD8^+^ T cell responses	Colorectal cancer, melanoma and pancreatic cancer cell lines and mice	[91]
GSTP1	Targeting GSTP1 in HEBP2‐overexpressing tumor cells reduces glutamine‐consumption‐dependent CCL3^+^ macrophage ferroptosis, thereby enhancing antitumor immunity	Breast cancer cell lines and mice	[189]
STING1	STING1 induces CD274 expression in tumor‐associated macrophages	Melanoma cell lines and mice	[81]
CD274 in tumor‐derived extracellular vesicles	CD274 antibody and reduce cholesterol synthesis	A375, A549, and MCF7 and B16‐F10 and A375/GP100 mouse	[205]
UGCG	Targeting the sphingomyelin synthase UGCG (with Eliglustat) induces CD8^+^ T cell dysfunction, reducing granzyme expression	Colorectal cancer or melanoma cell lines and mice	[93]
QDPR	*QDPR* deficiency creates an immunosuppressive microenvironment, whereas BH4 supplementation restores immunotherapy sensitivity	Pancreatic cancer and mice	[92]
*Fusobacterium nucleatum*	Clearing *Fusobacterium nucleatum*, significantly enhancing the efficacy of immune checkpoint inhibitors by activating CD8^+^ T cells	Colorectal cancer mice	[97]
Microbially derived butyrate	Directly activates CD8^+^ T cell function by suppressing inhibitor of ID2	Colorectal cancer mice	[98]
Short‐chain fatty acids from microorganisms	Prolonged progression‐free survival and improved clinical response rates following anti‐PDCD1 therapy	Various solid tumor patients	[206, 207, 208]
Butyrate and propionate	Elevated systemic butyrate and propionate levels correlate with CTLA4 blockade resistance and increased immunosuppressive Treg populations	Metastatic melanoma patients	[99]
Acetate	Dietary acetate supplementation promotes tumor growth and suppresses CD8^+^ T cell infiltration	NSCLC mice	[209]
*L. reuteri*	I3A released by *L. reuteri* activates the AHR within CD8^+^ T cells, promoting IFNG secretion and enhancing T‐cell effector function	Melanoma mice	[100]
PDCD1LG2	Suppresses T cell activity via the repulsive guidance molecule BMP co‐receptor B (RGMB) receptor	Colorectal cancer mice	[104]
*Burkholderia cepacia*, *Bacteroides fragilis*, and *Corynebacterium kangii*	Improve the immunosuppressive microenvironment and promote anti‐PDCD1 treatment	B16‐F10 melanoma mouse	[105]
PRDM12	PRDM12 induces CD8^+^ T cell exhaustion via the CGRP‐RAMP1 neuroimmune axis and H3K9me3 chromatin remodeling	Melanoma mice	[111]
ATF3 and IFNAR1	Tumor denervation achieved by knocking out the *ATF3* gene in neurons or blocking IFNAR1 enhances the efficacy of anti‐PD‐1 therapy	Advanced cutaneous squamous cell carcinoma and mice	[108]
GRIN2D	Sensory neurons release glutamate via pseudopodia, activating GRIN2D receptors to trigger calcium signaling and transcriptional reprogramming	PDAC cell lines and mice	[210]
GRM8	Cancer cells receive synaptic inputs mediated by NMDA receptors and GABAA receptors to promote proliferation	SCLC cell lines and mice	[112]
Serotonin	Platelets within the tumor microenvironment release serotonin to promote tumor growth	Pancreatic cancer, colorectal cancer cell lines and mice	[113]
Nivolumab (PDCD1i) plus ipilimumab (CTLA4i)	Coexpression of inhibitory receptors is common on severely exhausted T cells in the tumor. Targeting multiple inhibitory receptors might overcome resistance in the form of one inhibitory receptor compensating for another, when one is blocked	Various tumor patients	[114, 115, 116, 117, 118, 119]
Nivolumab with relatlimab (LAG3i)		Melanoma	[122]
PDCD1 inhibitors paired with HAVCR2, or TIGIT inhibitors		Various tumor patients	[123]
Pembrolizumab (PDCD1i) and INBRX‐106 (OX40 agonist)	Addition of agonistic antibodies to engage costimulatory molecules might help improve the effector functions of T cells. The coinhibitory receptor–costimulatory combination is equated to both taking one foot off the brake (coinhibitory receptor blockade) and putting the other foot on the gas (costimulation engagement)	The early stages of solid tumors	[124]
Pembrolizumab or spartalizumab (PDCD1i) combined with TRX518		Advanced solid tumors and/or lymphoma	[125, 126, 127]
PEGylated IL2 with nivolumab	Cytokines supplementation can lead to a more proinflammatory TME, leading to better activation of dendritic cells and uptake of tumor cell antigens	Metastatic melanoma, renal cell carcinoma, NSCLC, urothelial carcinoma, and triple‐negative breast cancer	[129]
ALKS4230 or with pembrolizumab		Various tumor patients	[130, 131]
GDF15	GDF15 aids tumors in evading immune attacks by interfering with T cell migration and activity	NSCLC and urothelial carcinoma cell lines and mice	[132]
Nivolumab plus chemotherapy	Enhanced tumor cell killing and antigen release. The efficacy of chemotherapy and immune checkpoint therapy is highly dependent on appropriate dosage, timing, and scheduling.	Advanced esophageal squamous cell carcinoma patients	[134]
Metronomic chemotherapy plus PDCD1i		metastatic breast cancer patients	[135]
Radiotherapy plus immunotherapy	Enhanced tumor cell killing and antigen release. Tumor volume reduction may confer a numerical advantage to the immune system. Timing and sequencing may be critical to success	Various tumor patients	[137, 138]

### 4.1 Tumor‐Intrinsic Adaptation and Therapy Strategies

Loss of immunogenic neoantigens, defects in MHC class I antigen presentation machinery (caused by mutations in HLA or B2M) [64, 65], and impairments in IFNγ signaling [66, 67, 68] represent key intrinsic mechanisms through which tumors evade immune recognition and acquire resistance to immunotherapy (Figure 3a). Conceptually, these tumor‐intrinsic alterations disconnect checkpoint blockade from effective immune killing: by limiting what immune cells can see (antigen availability) or how they can respond (IFNγ pathway integrity), they impose an upstream ceiling on the therapeutic benefit achievable through modulating inhibitory checkpoints alone. Accordingly, strategies aimed at restoring antigen presentation and IFNγ responsiveness—rather than merely intensifying checkpoint inhibition—are critical for overcoming this layer of resistance.

For instance, in MB49 bladder carcinoma and B16F10 melanoma models, inhibiting phosphatidylserine synthase 1 (PTDSS1) enhances HLA expression by activating the IFNG pathway, thereby boosting CD8^+^ T cell killing [69]. Similarly, in a melanoma vaccine trial, one initially responsive patient developed resistance due to *B2M* deficiency [65]. In contrast, high B2M expression correlates with poor prognosis in glioma, glioblastoma, and colorectal cancer [70, 71]. In colorectal cancer, this correlates with HLA‐E co‐expression, where HLA‐E binds to the inhibitory receptors CD94/killer cell lectin like receptor C1 (KLRC1, also known as NKG2A) on CD8^+^ T cells and NK cells, suppressing their cytotoxic activity [72]. Of note, using demethylating agents (e.g., 5‐azacytidine) or IFNG stimulation reverses resistance caused by HLA transcriptional suppression, reactivating antigen presentation [73].

In summary, overcoming tumor‐intrinsic resistance requires precise patient stratification based on molecular features—such as *HLA* and *B2M* deletion—and the development of rationally designed combination therapies that target these specific defects to optimize efficacy while managing toxicity. This, however, represents only one layer of the resistance landscape. Even when immune recognition is restored, effector function can remain constrained by a profoundly remodeled tumor microenvironment, underscoring the need to shift focus from tumor cell–centric mechanisms to the next layer of resistance: TME‐mediated suppression.

### 4.2 Microenvironmental Remodeling and Therapy Strategies

Beyond tumor‐intrinsic alterations, the tumor microenvironment (TME) constitutes a second critical layer of immunotherapy resistance. The TME is a dynamic ecosystem composed of abnormal vasculature, extracellular matrix components, diverse immune populations, and a complex milieu of secreted factors. Within this ecosystem, three interconnected features—immune heterogeneity, metabolic dysregulation, and immune cell senescence—represent core drivers of resistance, collectively limiting the infiltration, persistence, and effector function of antitumor immune cells. TME remodeling thus serves as a critical checkpoint that determines whether restored tumor recognition can successfully translate into sustained cytotoxic activity. In doing so, it directly links microenvironmental status to the durability of therapeutic response and the risk of relapse, underscoring the need for strategies that reprogram the TME rather than solely targeting tumor cells or checkpoints.

#### 4.2.1 Immune Cell Heterogeneity and Abnormalities

Immune cell heterogeneity and functional abnormalities within the TME drive resistance to immunotherapy (Figure 3b). For instance, the proportion of the CD56^dim^ CD16^hi^ subset in natural killer (NK) cells is reduced in lung and renal carcinomas, whereas breast and esophageal cancers exhibit an inverted NK cell distribution pattern. TANK cells (CD56^dim^ CD16^hi^ C6‐Dnajb1 subset) demonstrate the lowest toxicity and are associated with poor prognosis [74]. Pharmacologic inhibition of NFE2L2 with Brusatol, combined with anti‐PDCD1 therapy, decreased intratumoral TREM2^+^ macrophages, enhanced NK‐cell cytotoxicity, and restored T‐cell effector function in lung cancer models [75]. CAFs suppress T‐cell activity both directly through the PDCD1/CD274 axis and indirectly by upregulating CD274 on tumor cells, promoting immune escape in melanoma and colorectal cancer [76, 77]. However, targeted elimination of actin alpha 2, smooth muscle (ACTA2; also known as α‐SMA) CAFs paradoxically accelerated tumor growth in pancreatic cancer mice and increased infiltration of immunosuppressive cells within the tumor microenvironment, indicating functionally distinct CAF subsets with opposing roles [78]. In addition, tumor‐secreted AE binding protein 1 (AEBP1) binds to the cytoskeleton associated protein 4 (CKAP4) receptor on CAF surfaces, activating the AKT serine/threonine kinase (AKT)‐CD274 signaling pathway and subsequently inducing T‐cell functional exhaustion. This mechanism is crucial for immune evasion in colorectal adenocarcinoma and triple‐negative breast cancer [79].

Current strategies targeting these mechanisms include: 1) Targeting CAFs to remodel the physical barrier: For example, Polyethylene glycol‐modified recombinant human hyaluronidase (PEGPH20) degrades hyaluronic acid in combination with protein tyrosine kinase 2 (PTK2) inhibitors, enhancing the efficacy of anti‐PDCD1 therapy in mouse pancreatic cancer [80]. 2) Blocking immunosuppressive signals: Combining toll like receptor 2 (TLR2) agonist (Pam3CSK4) with STING1 agonist (DMXAA) reverses CD274 upregulation in macrophages, enhancing melanoma treatment efficacy [81]. 3) Reversing immunosenescence: Senolytic drugs can restore the immune response of a variety of solid tumors [82, 83].

In summary, developing multi‐target combination strategies holds promise for overcoming immune‐heterogeneous resistance. These include simultaneously targeting CAF subpopulation‐specific signaling pathways—such as the secreted phosphoprotein 1 (SPP1)‐DAN family BMP antagonist (GREM1)‐ morphogenetic protein 2 (BMP2) circuit [84] alongside T cell exhaustion programs and axes like CXCL16‐CXCR6 [85]. However, beyond cellular composition and stromal barriers, the metabolic landscape of the tumor microenvironment imposes additional constraints on effector cells. This underscores that metabolic checkpoints represent a key, and potentially orthogonal, layer of regulation that must be addressed to fully unleash the efficacy of immunotherapy.

#### 4.2.2 Metabolic Abnormalities in the TME

Metabolic abnormalities drive immunosuppression through nutrient competition and accumulation of toxic metabolites (Figure 3c). For instance, tumor cells upregulate phospholipase A2 group X (PLA2G10) to produce lipid metabolites that inhibit T cell migration and NK cell infiltration. Elevated serum PLA2G10 levels in NSCLC patients correlate with resistance to anti‐PDCD1 therapy [86]. Polyamines promote Treg immunosuppressive phenotypes via the casein kinase II (CK2) pathway, whereas Treg‐specific casein kinase 2 beta (*CSNK2B*) knockout reverses this effect and enhances CD8^+^ T cell killing in B16‐F10 melanoma and MC38 colon cancer models [87]. In addition, pancreatic CAFs secrete acetate to regulate polyamine metabolism to promote tumor growth. Mechanistically, acetate induces lysine acetylation modification of sp1 transcription factor (SP1) via acyl‐CoA synthetase short chain family member 2 (ACSS2), enhancing its stability and subsequently triggering spermidine/spermine N1‐acetyltransferase 1 (SAT1)‐dependent polyamine metabolism [88]. Tumor cells can also internalize lysine and tryptophan via dihydroorotate dehydrogenase (Quinone) (DHODH)‐dependent macropinocytosis to produce succinyl‐CoA, inducing K120 succinylation of the MHC II transactivator (CIITA) and suppressing MHC II transcription, enabling immune evasion. This mechanism has been validated in A549, PANC‐1, HCT116, T‐47D, and LLC cells and murine breast cancer models [89]. Conversely, succinate enhances CD8^+^ T cell adaptability through BCL2 interacting protein 3 (BNIP3)‐mediated mitophagy and stemness gene activation, improving survival and synergizing with anti‐CD274 therapy in B16 and MC38 tumors. Clinically, elevated succinate levels in melanoma and gastric cancer patients correlate with improved immunotherapy efficacy [90].

Strategies targeting metabolic pathways include: 1) Intervening in nutrient competition: Inhibiting the lactate receptor hydroxycarboxylic acid receptor 1 (HCAR1) with reserpine reduces C‐C motif chemokine ligand 2 (CCL2)/7 secretion and PMN‐MDSC recruitment via the tyrosine 3‐monooxygenase/tryptophan 5‐monooxygenase activation protein zeta (YWHAZ)‐STAT3 pathway, enhancing PDCD1 antibody efficacy in colorectal cancer [7]; 2) Reconfiguring metabolic pathways: Inhibiting the lactate‐mediated MLX interacting protein (MLXIP)–thioredoxin interacting protein (TXNIP) signaling axis enhances CD8^+^ T cell responses and synergistically suppresses tumors in colorectal cancer, melanoma, and pancreatic cancer mice when combined with anti‐PDCD1 [91]; 3) Reversing immunosuppressive metabolites: tetrahydrobiopterin (BH4) supplementation restores immunotherapy sensitivity in pancreatic cancer [92]. However, certain metabolic interventions‐such as inhibiting UDP‐glucose ceramide glucosyltransferase (UGCG) with eliglustat‐impair CD8^+^ T cell granzyme expression, leading to treatment failure in colorectal cancer and melanoma [93].

In summary, overcoming metabolic resistance will require spatially and temporally integrated strategies—from single‐cell metabolic profiling to sequential therapies that target both nutrient deprivation and immunosuppressive metabolites. However, resistance extends beyond the local tumor microenvironment. Systemic host factors, including metabolic hormones, gut microbiota, and neuroimmune circuits, modulate immune tone from outside the tumor, ultimately shaping therapeutic dependence and clinical outcomes.

### 4.3 Host Immune Dysregulation and Therapy Strategies

Beyond the tumor microenvironment, systemic host factors—including the gut microbiota and neuroimmune networks—play a critical role in shaping antitumor immunity. This extratumoral layer of regulation helps explain why patients with similar tumor‐intrinsic features can exhibit divergent responses to immune checkpoint blockade, and why resistance may arise from cues that reinstate T cell exhaustion programs from outside the tumor. The gut microbiota can either potentiate or suppress immune responses, while the neuro–immune–tumor axis integrates neuronal and immune signaling to influence cancer progression. Together, these systemic factors extend the concept of immune regulation beyond the local microenvironment, adding a crucial dimension to our understanding of therapeutic resistance and response heterogeneity.

#### 4.3.1 Microbiome and Therapy Strategies

The gut microbiome profoundly influences immune therapy responses through metabolic signaling and immune modulation, but its dysregulation can lead to drug resistance (Figure 3d). For instance, in non‐responders, *Escherichia* enrichment and phenylacetylglutamine (PAGln) inhibit CD8^+^ T cell function via adrenergic receptors, reducing ICB efficacy in colorectal cancer models [94]. Caspase recruitment domain family member 9 (*Card9^−/−^*) deficiency triggers *Candida tropicalis* overgrowth, inducing myeloid cell differentiation into granulocyte‐like MDSCs (G‐MDSCs) that suppress T cell function and accelerate colon cancer progression [95]. *Helicobacter pylori* down‐regulates the killer cell lectin like receptor K1 (KLRK1) receptor of NK cells to promote gastric cancer immune escape [96]. Additionally, chronic stress induces CD8^+^ T cell exhaustion via the kynurenine‐ aryl hydrocarbon receptor (AHR) axis, accelerating head and neck squamous cell carcinoma progression.

Current strategies targeting these mechanisms include: 1) Targeting pathogenic bacteria: Liposomal delivery systems eliminate *Fusobacterium nucleatum*, restoring CD8^+^ T cell activity in colorectal cancer models [97]; 2) Metabolite regulation: Butyrate supplementation activates CD8^+^ T cells to enhance anti‐PDCD1 efficacy in colorectal cancer mice [98], though caution is warranted as it may induce T_reg_ expansion in melanoma [99]; Engineered probiotics like *L. reuteri* activate AHR via indole‐3‐carboxaldehyde (I3A), promoting IFNG secretion and enhancing T cell function in melanoma [100]. And metabolites such as L‐arginine, gut microbe‐derived trimethylamine N‐oxide (TMAO), and microbial vitamin B5 play critical roles in immunotherapy for distinct cancers, including multiple myeloma and breast cancer [101, 102, 103]; 3) Interfering with immune checkpoints: Specific microbiota (e.g., *Coprobacillus cateniformis*) suppress programmed cell death 1 ligand 2 (PDCD1LG2) expression, enhancing antitumor immunity in colorectal cancer models [104]. Furthermore, intratumoral injection of specific bacteria (*Burkholderia cepacia*, *Bacteroides fragilis*, and *Corynebacterium kangii*) combined with anti‐PDCD1 therapy significantly suppressed tumor growth in a B16‐F10 melanoma mouse model, increasing the ORR to anti‐PDCD1 treatment from 25% to 70% [105].

In summary, future personalized interventions incorporating microbiome sequencing‐such as dietary or prebiotic adjustments based on short‐chain fatty acid profiles (acetate, propionate, etc.)‐will optimize immune activation while avoiding metabolite‐content‐dependent side effects.

#### 4.3.2 Neuro‐Immune Interaction and Therapy Strategies

The neuroimmune axis drives immunosuppression and drug resistance through neurotransmitters, cytokines, and direct neuro‐tumor interactions (Figure 3e). Resistance mechanisms include: sympathetic nerves releasing norepinephrine via ADRB1 receptors to suppress CD8^+^ T cell and DC function, promoting T cell exhaustion in pancreatic cancer models [31]. Injured neurons releasing CGRP inhibit IL15 secretion by CAFs, weakening NK cell antitumor activity and accelerating PDAC progression [106]. Furthermore, PDAC reprograms neurons to express vascular endothelial growth factor A (*VEGFA*), matrix metalloproteinases (*MMPs*) via IL6 and nerve growth factor (NGF), promoting angiogenesis and immune suppression [107]. In cutaneous squamous cell carcinoma, injured neurons release IL6 and type I interferon to recruit M2 macrophages and exhaust CD8^+^ T cells, leading to anti‐PDCD1 resistance [108]. Sterile alpha and TIR motif containing 1 (SARM1)‐dependent axonal death induces Wallerian degeneration, activating neuroinflammatory responses, which promote the progression of glioblastoma [109]. In breast cancer, the neuropeptide substance P induces a small population of cancer cell apoptosis via the tachykinin receptor 1 (TACR1) receptor, releasing ssRNA that activates toll like receptor 7 (TLR7)‐driven non‐canonical phosphatidylinositol‐4,5‐bisphosphate 3‐kinase (PIK3)‐AKT pathways to promote metastasis [110].

Current overcoming strategies focus on disrupting neuroimmune suppression signals: 1) Targeting key pathways: Knocking out the PR/SET domain 12 (*PRDM12*) gene blocks the CGRP‐ modifying protein 1 (RAMP1) axis and H3K9me3 remodeling, reversing CD8^+^ T cell exhaustion in melanoma models [111]; 2) Intervening neural reprogramming: Tumor denervation (e.g., neuronal *ATF3* or interferon alpha and beta receptor subunit 1 [IFNAR1] knockout) enhances anti‐PDCD1 efficacy in cutaneous squamous cell carcinoma [108]; 3) Inhibiting neurotransmitter signaling: Targeting glutamate pathways (e.g., glutamate metabotropic receptor 8 (GRM8) receptor inhibitor DCPG or glutamate release inhibitor Riluzole) suppresses tumor invasion in PDAC and small cell lung cancer models [112]; 4) Combination with neuromodulatory drugs: Serotonin inhibitors (e.g., fluoxetine) combined with anti‐PDCD1 therapy suppress growth in pancreatic and colorectal cancer models [113].

In summary, future strategies should integrate spatiotemporally precise interventions—such as locoregional delivery to minimize neurotoxicity—with multi‐omics profiling to identify predictive neuroimmune biomarkers. Tumor‐intrinsic defects, local microenvironmental barriers, and systemic host factors converge to form a multilayered immune evasion network. Rational combination therapy must therefore target complementary nodes within this network to relieve sequential bottlenecks and achieve durable responses.

### 4.4 Combination Therapy Strategy

The multilayered immune evasion mechanisms operating within the tumor microenvironment collectively limit the clinical efficacy of immunotherapy. Multi‐target synergistic combination strategies that block complementary resistance pathways represent a rational approach to overcoming these limitations and expanding the patient population that benefits from treatment. In practice, this requires mapping clinical non‐response or relapse back to the dominant limiting layers—whether checkpoint signaling, metabolic suppression, antigen presentation defects, myeloid/stromal remodeling, or systemic dysregulation—and then assembling mechanistically coherent combinations that address these specific constraints.

Current combination approaches encompass several major modalities:

1) Dual immune checkpoint blockade. Combinations of ICIs have achieved notable clinical success. The co‐administration of nivolumab (PDCD1 inhibitor) and ipilimumab (CTLA4 inhibitor) is approved for multiple cancers—including melanoma, metastatic cervical cancer, hepatocellular carcinoma, NSCLC, renal cell carcinoma (RCC), and microsatellite instability–high (MSI‐Hi) colorectal cancer [114, 115, 116, 117, 118, 119]. However, this combination shows limited efficacy in glioblastoma and pancreatic cancer [120, 121], suggesting that tumor‐specific differences in PDCD1/CTLA4 combined responses stem from complex interactions between tumor cells and TME. Furthermore, the combination of nivolumab with relatlimab (a LAG3 inhibitor) has been approved for melanoma treatment [122]. Different combinations—such as PDCD1 inhibitors paired with HAVCR2, or T cell immunoreceptor with Ig and ITIM domains (TIGIT) inhibitors—demonstrate varying efficacy across different cancers. This may be partly attributed to LAG3 influencing the early stages of T cell activation and priming, whereas TIGIT may play a greater role in suppressing the function of Tregs and tolerogenic dendritic cells [123]. It may also be partly attributable to subject variability and other factors.

2) Co‐stimulatory agonists with ICIs. The mechanism of action for the combination of inhibitory signals with co‐stimulatory signals involves blocking inhibitory signals while simultaneously activating co‐stimulatory pathways. Classic examples such as Pembrolizumab (PDCD1i) and INBRX‐106 (OX40 agonist) have demonstrated safety and efficacy in the early stages of solid tumors [124]. Pembrolizumab or spartalizumab (PDCD1i) combined with TRX518 (TNF receptor superfamily member 18 [TNFRSF18] agonist) has also been approved for clinical trials in advanced solid tumors and/or lymphoma [125, 126, 127].

3) Cytokine‐based combinations. Cytokine combination therapy enhances treatment efficacy by boosting T‐cell activity or inducing an inflammatory microenvironment. Traditional IL‐2 (Aldesleukin) exhibits a short half‐life and high toxicity [128]. In contrast, the combination therapy of engineered cytokine bempegaldesleukin (PEGylated IL2) with nivolumab demonstrated favorable safety in a phase I clinical trial. This study encompassed five solid tumors: metastatic melanoma, renal cell carcinoma, NSCLC, urothelial carcinoma, and triple‐negative breast cancer [129]. Similarly, the engineered cytokine ALKS4230 (Nemvaleukin alfa) selectively activates CD8^+^ T cells and NK cells while inhibiting Treg proliferation. Its efficacy as a monotherapy or in combination with pembrolizumab for treating solid tumors is currently under evaluation [130, 131]. Additionally, the growth differentiation factor 15 (GDF15) neutralizing antibody visugromab combined with nivolumab overcomes CD274‐mediated treatment resistance in NSCLC and urothelial carcinoma in the GDFATHER‐1/2a clinical trial [132]. It should be noted that GDF15 is known to regulate appetite and body weight. In some trials, patients with high baseline GDF15 levels experienced weight gain following treatment. Whether systemic neutralization of GDF15 may induce other metabolic issues remains unclear [133].

4) ICIs with conventional therapies. Combining ICIs with chemotherapy or radiotherapy can enhance tumor antigen release, augment T‐cell activation, and remodel the TME. However, such benefits are highly context‐dependent. For example, nivolumab plus chemotherapy significantly extended survival in advanced esophageal squamous cell carcinoma [134], and metronomic chemotherapy (cyclophosphamide + capecitabine + vinorelbine) combined with PDCD1 blockade improved survival in metastatic breast cancer [135]. Yet, not all combinations yield superior outcomes: in the phase III IMvigor130 trial for metastatic urothelial carcinoma, atezolizumab plus platinum‐based chemotherapy did not improve overall survival in the intention‐to‐treat population [136]. Similarly, radiotherapy combined with immunotherapy also exhibits heterogeneous efficacy [137, 138]. This heterogeneity may stem from the complex interplay of drug interactions, tumor microenvironment, and host factors. Currently, combination targeted therapies (such as tyrosine kinase inhibitors) and vaccines are also being explored in clinical trials across multiple cancer types.

Multimechanistic combination immunotherapies have shown promise in select cancers, but realizing their full potential requires standardized biomarkers to guide patient selection and monitor immune dynamics. Beyond drug combinations, regulated cell death modalities represent an additional mechanistic layer within the tumor–immune network. By governing antigen release, danger signals, and the immunostimulatory versus tolerogenic balance, distinct cell death pathways can fundamentally shape both ICB sensitivity and resistance.

## 5 Cell Death in Cancer Immunotherapy

**FIGURE 4:**
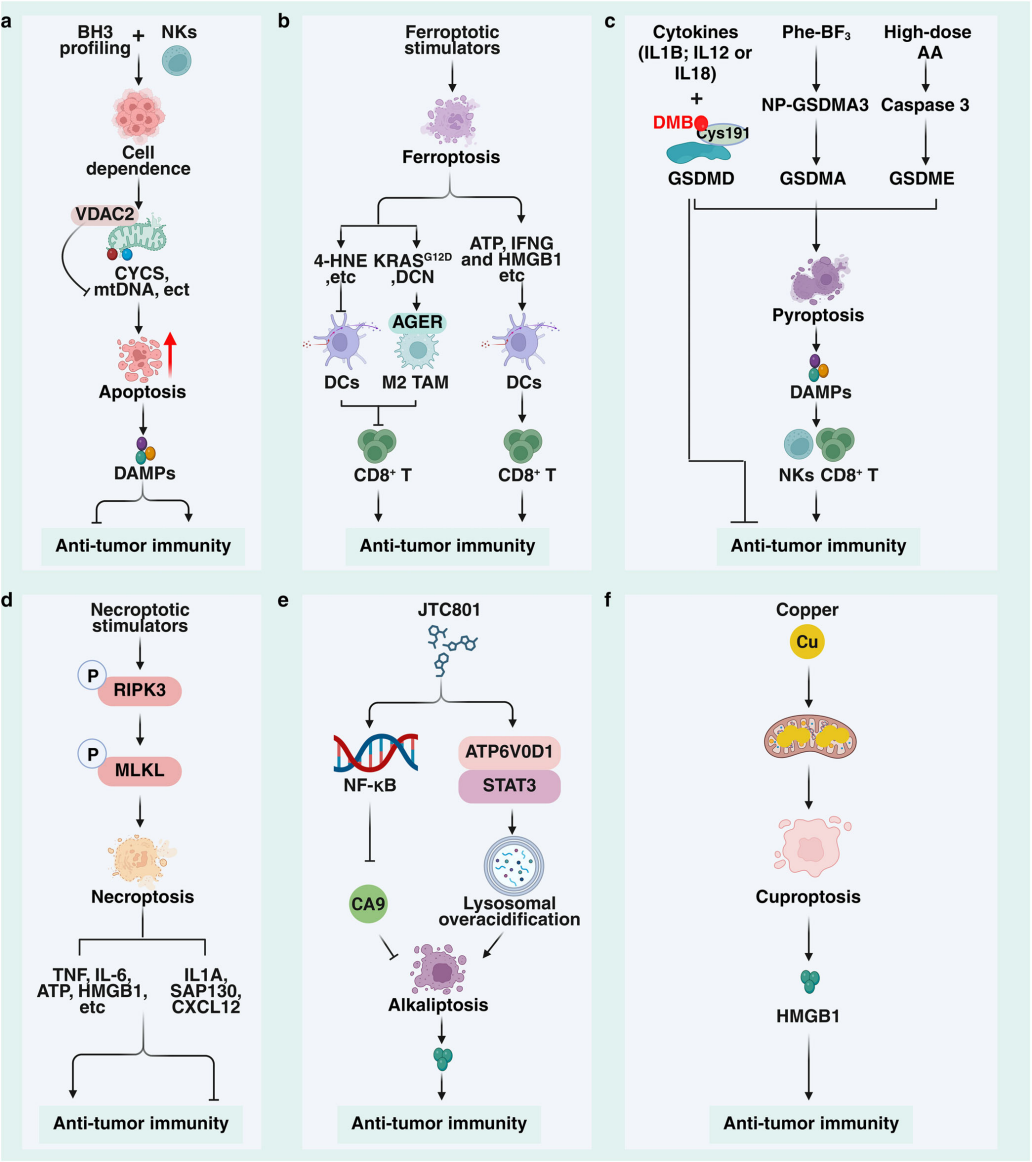
Cell death in cancer immunotherapy. (a) BH3 analogues combined with NK cells induce mitochondrial‐dependent apoptosis, releasing cytochrome c (CYCS) and mtDNA to enhance immune responses, while VDAC inhibits the release of CYCS and mtDNA. Furthermore, different DAMPs released during apoptosis exert dual roles in antitumor immunity. (b) Ferroptosis exhibits dual properties in tumor immunotherapy. On one hand, ferroptosis induced by different stimuli can overcome tumor resistance while releasing DAMPs such as HMGB1, ATP, and IFNG, which promote antigen presentation by dendritic cells and enhance CD8^+^ T cell function. Conversely, lipid substances like 4‐HNE released by ferroptotic cells suppress antigen presentation. Additionally, ferroptotic cells release KRAS^G12D^ and DCN molecules, which bind to AGER receptors on M2 macrophages, thereby suppressing CD8^+^ T cell function. (c) Pyroptosis exhibits dual advantages in tumor immunotherapy. Phe‐BF3 releases GSDMA3 from NP‐GSDMA3, activating GSDMA to induce pyroptosis in cancer cells. High‐dose ascorbic acid (AA) induces pyroptosis via the ROS‐caspase3‐GSDME pathway. Cytokine combinations further amplify antitumor immune effects; for instance, GSDMD variants combined with IL1B/IL12/IL18 enhance antitumor activity. The small molecule DMB induces pyroptosis by binding to the Cys191 site of GSDMD, activating CD8^+^ T cells and NK cells. Pyroptotic cells also release DAMPs to further enhance antitumor immunity. However, GSDMD may also suppress the therapeutic effects of PD‐1 blockade. (d) Necroptosis exhibits dual characteristics in anti‐tumor immunotherapy. On one hand, RIPK3‐dependent necroptotic tumor cells release various inflammatory mediators such as TNF, IL‐6, ATP, HMGB1 etc., to promote antigen‐presenting cell (APC) maturation and CD8^+^ T cell activation, thereby amplifying the immune response. On the other hand, necroptotic cells may also contribute to an immunosuppressive microenvironment by releasing immunosuppressive factors like IL1A and CXCL12 or by recruiting myeloid suppressor cells via the SAP130 pathway. (e‐f) Alkaliptosis and cuproptosis, as novel forms of regulated cell death, release DAMPs such as HMGB1, potentially enhancing antitumor immunity.

The ultimate goal of cancer therapy is to selectively eliminate malignant cells while sparing normal tissues, with a central focus on modulating regulated cell death pathways. Distinct forms of cell death can exert dual—either immunostimulatory or immunosuppressive‐effects on antitumor immunity [139, 140]. Precisely inducing immunogenic cell death (ICD) while simultaneously suppressing tolerogenic or immunosuppressive signals can markedly enhance the efficacy of immunotherapy. Within this framework, regulated cell death represents an execution layer that links direct tumor killing to immune remodeling. Its relevance to cancer immunotherapy lies not only in cytotoxicity itself, but also in its capacity to alter antigen release, inflammatory signaling, dendritic‐cell activation, and immune‐cell recruitment, thereby shaping the magnitude and durability of responses to immune checkpoint inhibitors (Figure 4).

### 5.1 Apoptosis

Apoptosis has traditionally been considered non‐immunogenic. However, emerging evidence demonstrates that precise mechanistic regulation can convert apoptosis into an immunogenic form of cell death, opening new avenues for targeted cancer therapy (Figure 4a). In this context, apoptosis can be viewed as a “tunable” immunogenicity module: depending on the extent of DAMP release and immune sensing, the same death pathway may either reinforce antitumor immunity or fail to sustain it, thereby shaping both immunotherapy sensitivity and the risk of relapse.

For example, BH3 analogues such as S63845 and ABT‐199, when combined with natural killer (NK) cells, synergistically induce apoptosis in OCI‐AML3 and HeLa cancer cells at the mitochondrial level, achieving potent tumor suppression in mouse models [141]. Similarly, targeting the mitochondrial protein voltage dependent anion channel 2 (VDAC2) enhances the sensitivity of B16‐OVA, MC38, and LOVO tumor cells to CD8^+^ T cell and IFNG therapy. This mechanism involves activating BCL2 antagonist/killer 1 (BAK1) to promote cytochrome c (CYCS) release, thereby triggering apoptosis. Concurrently, mitochondrial DNA leakage activates the cGAS‐STING1 pathway to drive the immune response [142]. Notably, cytosolic CYCS can inhibit ferroptosis in PANC1 and HT‐1080 cells, suggesting that CYCS exerts context‐dependent functions across different cell death modalities [143].

In colorectal cancer cells (SW480 and CT26), oxaliplatin and 5‐fluorouracil (5‐FU) treatment induces the release of high‐mobility group box 1 (HMGB1) and heat shock protein family A member 4 (HSPA4), which enhance T‐cell activation through dendritic cell (DC) stimulation via TLR signaling [144]. Paradoxically, 5‐FU treatment also promotes apoptotic escape via the ATP‐Purinergic receptor P2×4 (P2RX4)‐mechanistic target of rapamycin kinase (MTOR) pathway, and inhibiting this pathway enhances antitumor efficacy [145]. Such divergent outcomes likely reflect variations in epigenetic states, treatment regimens, DAMP profiles, and tumor models. A key practical implication of apoptotic pathway regulation is illustrated in colorectal cancer, where chemotherapy‐induced apoptosis can simultaneously enhance DC–TLR‐driven T cell activation via the HMGB1–HSPA4 pathway and promote apoptotic escape through the ATP–P2RX4–mTOR axis [144, 145]. This duality underscores that therapeutic efficacy depends on the net balance between immune stimulatory output and apoptosis escape mechanisms induced by a given regimen. Accordingly, apoptosis‐directed combination strategies may be particularly relevant in the following contexts: (i) settings where NK/T cell effector function can be potentiated (e.g., NK cell activity against OCI‐AML3/HeLa lines); (ii) tumor models exhibiting IFNγ‐mediated immune activation (B16‐OVA/MC38/LOVO); and (iii) chemotherapy‐based colorectal cancer models characterized by distinct DAMP profiles and apoptotic escape pathways (SW480/CT26).

In summary, apoptosis is not a passive or immunologically silent process. Its impact on cancer immunotherapy is determined by the nature and magnitude of DAMP release and the subsequent immune sensing. When appropriately modulated—particularly through rationally designed combination regimens—classical apoptosis can be converted into immunogenic apoptosis, thereby amplifying therapeutic efficacy. This concept of “immunogenicity conversion” directly informs combination therapy design: even in the presence of checkpoint inhibitors, insufficient immune priming or inadequate danger signaling may still limit responses. By strategically regulating apoptotic pathways, antigen presentation and T cell activation can be enhanced, effectively overcoming such bottlenecks.

### 5.2 Ferroptosis

Ferroptosis plays a dual role in tumor immune regulation, and its targeted induction has emerged as a promising strategy to enhance immunotherapy efficacy (Figure 4b) [146]. By modulating lipid peroxidation, oxidative stress, and nutrient availability, ferroptosis influences both tumor cell vulnerability and immune cell adaptability. This positions ferroptosis as a mechanistic bridge linking metabolic checkpoint regulation to immunotherapy responsiveness.

On one hand, IFNG secreted by activated immune cells such as T cells can downregulate cystine/glutamate antiporter system xc^−^ (SLC3A2 and SLC7A11) expression or upregulate acyl‐CoA synthetase long‐chain family member 4 (ACSL4) in tumor cells. This synergistically induces ferroptosis in tumor cells through specific fatty acids within the tumor microenvironment in models such as ovarian cancer, thereby enhancing antitumor immunity [147, 148, 149] Of note, IFNG is produced not only by CD8^+^ and Th1 cells but also by NK and NKT cells, indicating that various immune subsets can trigger ferroptosis in lipid‐rich TMEs through IFNG‐dependent mechanisms [147, 148].

Conversely, ferroptosis can also impair antitumor immunity under certain conditions [150, 151]. Persistent activation of x‐box binding protein 1 (XBP1) in tumor‐associated dendritic cells (DCs) induces aberrant triglyceride synthesis through lipid peroxidation products such as 4‐hydroxynonenal (4‐HNE), leading to lipid accumulation, defective antigen presentation, and immune evasion in ovarian cancer [152]. Similarly, oxygenated lipid mediators released from ferroptotic polymorphonuclear myeloid‐derived suppressor cells (PMN‐MDSCs) suppress T‐cell function and reduce immunotherapy efficacy in lymphoma, colorectal cancer, and lung cancer models [153]. Tumor cells themselves can develop ferroptosis tolerance through metabolic adaptation—for example, by importing itaconate via solute carrier family 13 member 3 (SLC13A3) to neutralize lipid peroxidation [154]. In CD8^+^ T cells, CD36‐mediated fatty acid uptake induces lipid peroxidation and exhaustion, thereby diminishing the response to anti‐PDCD1 therapy in melanoma and multiple myeloma [155]. Moreover, ferroptotic PDAC cells release KRAS proto‐oncogene, GTPase (KRAS)^G12D^ protein, which polarizes macrophages toward an immunosuppressive phenotype through autophagy‐dependent mechanisms [156]. Of note, the immunogenicity of ferroptosis remains an area of active debate. Although ferroptotic cells can release content‐dependent DAMPs [157, 158, 159, 160].

In summary, when coupled with IFN‐γ‐driven tumor fragility—such as through systemic Xc^−^ inhibition or ACSL4 induction—ferroptosis can amplify immune‐mediated killing. However, depending on the lipid metabolic landscape and myeloid composition of the microenvironment, it may also exert immunosuppressive effects by impairing antigen presentation and promoting effector cell exhaustion. These dual roles manifest in tumor type–specific contexts: ovarian cancer models illustrate the benefit of IFN‐γ–coupled tumor ferroptosis, whereas lipid accumulation in dendritic cells (XBP1/4‐HNE) and PMN‐MDSC–derived mediators in lymphoma, colorectal, and lung cancer models highlight scenarios where ferroptosis‐associated lipid signaling suppresses immunity and compromises immunotherapy efficacy. This divergence likely reflects variations in tumor heterogeneity, microenvironmental lipid composition, inducer specificity, and the timing of cell death [150]. Thus, the relevance of ferroptosis to ICI efficacy lies in its context‐dependent immunological output. When appropriately induced in tumor cells, ferroptosis may enhance inflammatory signaling and increase tumor vulnerability to T‐cell‐mediated attack; however, uncontrolled lipid peroxidation may also compromise antitumor immunity. Therefore, ferroptosis‐based combinations with ICIs should be considered in a context‐, timing‐, and cell‐specific manner.

### 5.3 Pyroptosis

Targeted and controllable therapies based on pyroptosis aim to precisely induce this highly inflammatory form of cell death to eliminate tumor cells and activate antitumor immunity, while simultaneously limiting the risk of excessive inflammation (Figure 4c) [161, 162]. Compared with apoptosis and ferroptosis, pyroptosis represents a distinct network trade‐off: amplifying inflammatory signaling may enhance immunotherapy responses, but uncontrolled inflammation can also remodel the tumor microenvironment in ways that undermine durable benefit. Thus, “controllability” is central to the therapeutic design of pyroptosis‐based strategies.

For example, GSDMA3 can specifically induce pyroptosis in HeLa (cervical cancer) and EMT6/4T1 (breast cancer) cells, while nanoparticle‐delivered GSDMA3 enhances antitumor efficacy in mice [163]. Similarly, in lung cancer cells harboring the serine/threonine kinase 11 (*STK11*) mutation, high‐dose ascorbic acid can trigger pyroptosis via reactive oxygen species (ROS)‐caspase 3‐GSDME pathway, thereby synergistically enhancing the efficacy of anti‐PD‐1 therapy [164]. In models of B16‐F10 melanoma, 4T1 breast cancer and CT26 colorectal cancer, engineered GSDMD variants combined with cytokines such as IL1B, IL12, or IL18 significantly enhanced immune activation and tumor control. However, IL1B and IL18 may have a negative effect on protective effects via IFNG‐dependent immunosuppressive pathways, highlighting the importance of selecting the correct cytokines and controlling doses for safety and efficacy [165]. Conversely, *GSDMD* deficiency may enhance melanoma response to CD274 therapy [166], highlighting the complex, context‐dependent role of GSDMD in immune regulation.

In summary, future efforts should focus on developing precision delivery systems and controlled activation strategies for pyroptosis to maximize therapeutic efficacy while minimizing systemic toxicity. As discussed in the models above, pyroptosis can function as an “immune activation amplifier”‐significantly enhancing responses to anti‐PD‐1 therapy in STK11‐mutant lung cancer or intensifying tumor control in HeLa, EMT6, and 4T1 models. However, its net effect is context‐dependent, shaped by the accompanying inflammatory microenvironment, including cytokine profiles and IFN‐γ‐related antagonistic signals. It has a more direct conceptual link to ICI sensitization than cell death modalities that are relatively immunologically silent. Its therapeutic value lies in its potential to promote immune‐cell recruitment and local activation, although this benefit must be balanced against the risk of excessive inflammatory toxicity.

### 5.4 Necroptosis

Necroptosis, a form of programmed cell death, offers novel therapeutic targets for overcoming tumor resistance to apoptosis and modulating anti‐tumor immunity through its the receptor‐interacting serine/threonine kinase 1 (RIPK1)–RIPK3–mixed lineage kinase domain‐like pseudokinase (MLKL) signaling cascade [167]. However, its dual immunomodulatory effects‐both immune activation and suppression‐necessitate controlled induction (Figure 4d). In the resistance framework, necroptosis is particularly relevant because it can bypass apoptosis resistance while simultaneously reshaping myeloid recruitment and cytokine milieus—two determinants repeatedly implicated in TME‐driven failure of immunotherapy.

For example, in the B16‐F10‐OVA melanoma and LL/2‐OVA adenocarcinoma models, RIPK3‐dependent necroptosis significantly enhances antitumor immunity [168]. Conversely, *Ripk3* or *Mlkl* deficiency attenuates antitumor immune responses in EO771 and MC38 tumor‐bearing mice, accompanied by reduced IFNG and TNF production [169]. Conversely, necroptosis can also suppress antitumor immunity by releasing signals such as IL1A (in Lewis lung cancer and MC38 colon cancer) to recruit immunosuppressive myeloid cells, or by promoting an immunosuppressive microenvironment through the CXCL1‐ C‐type lectin domain family 4 member E (CLEC4E) axis (as in pancreatic cancer) and the poly(ADP‐Ribose) polymerase 1 (PARP1)‐MLKL‐CXCL12 pathway (in ovarian and breast cancer), these mechanisms instead constrain T cell function and lead to therapeutic resistance [170, 171, 172]. A key practical implication of necroptosis is its context‐dependent role in immunotherapy. When apoptotic resistance predominates and downstream inhibitory signals—such as IL‐1A‐mediated recruitment—can be synergistically targeted, necroptosis‐based strategies may prove most effective. Conversely, in tumor microenvironments where necroptosis‐associated chemokines and cytokines promote myeloid suppression—as observed in pancreatic, ovarian, and breast cancers—unrestrained induction of necroptosis may paradoxically exacerbate resistance to immune checkpoint blockade.

Therefore, the key to designing therapies targeting necroptosis lies in precisely regulating its induction (e.g., specific activators or nanocarrier delivery) and intervening in its downstream immune signaling (e.g., neutralizing IL‐1A). This approach maximizes its immune‐stimulatory potential while mitigating its potential pro‐inflammatory and immunosuppressive risks.

### 5.5 Other cell death modalities

Accumulating evidence indicates that several emerging forms of regulated cell death may also exhibit immunogenic properties, thereby contributing to antitumor immunity. These emerging modalities extend the network map of regulated cell death and suggest that distinct biochemical stress programs (e.g., pH imbalance or metal‐dependent mitochondrial stress) may converge on shared immune‐relevant outputs such as DAMP release, thereby offering additional opportunities to modulate immunotherapy responsiveness.

Alkaliptosis represents a noncanonical, pH‐dependent form of regulated cell death driven by disrupted cytoplasmic–lysosomal pH homeostasis [173, 174, 175]. Treatment with JTC801, a known alkaliptosis inducer, triggers the release of DAMPs such as HMGB1, implicating its potential immunogenic nature; however, in vivo vaccination or tumor regression studies are still lacking to confirm these effects (Figure 4e) [176].

Cuproptosis is a copper‐dependent mode of mitochondrial stress–induced cell death characterized by proteotoxic aggregation of lipoylated enzymes and loss of iron–sulfur cluster proteins [177]. Cuproptotic Calu1 cells release HMGB1 [178], a hallmark DAMP associated with classical inflammatory cell death. Consistently, nanosystem‐induced cuproptosis markedly enhances inflammation within the tumor microenvironment, leading to potent antitumor effects in 4T1 breast cancer and colorectal cancer mouse models [179, 180]. Mechanistically, cuproptosis may partially overlap with ferroptosis [181], suggesting potential convergence between metal‐dependent oxidative stress and immunogenic cell death pathways (Figure 4f).

In addition, emerging cell death pathways such as triaptosis and mitoxyperiosis have shown antitumor potential in preclinical models [182, 183], though their underlying immune‐modulatory mechanisms remain largely unexplored. Thus, their potential relevance lies in whether can be harnessed to trigger tumor‐selective stress responses that favor immune activation. At present, their roles are best framed as a promising but still exploratory component of ICI‐oriented combination design.

Taken together, different cell deaths differ in their immunological output, but all may influence ICI efficacy by altering antigen availability, innate immune activation, T‐cell recruitment, and the inflammatory tone of the tumor microenvironment. The key translational question is therefore not which death mode is universally superior, but which modality, in which tumor context, can most effectively relieve the dominant bottleneck limiting checkpoint blockade.

## 6 Multimodal Technology Optimizes Anti‐Tumor Immunotherapy

The fourth layer of the framework is therapeutic optimization. Rational immunotherapy should not rely on empirical combination alone, but should match specific interventions to the dominant barrier operating in a given tumor state. This section therefore focuses on how mechanistic stratification, dynamic biomarkers, and appropriately timed combinations may improve the efficacy and durability of ICIs

**FIGURE 5:**
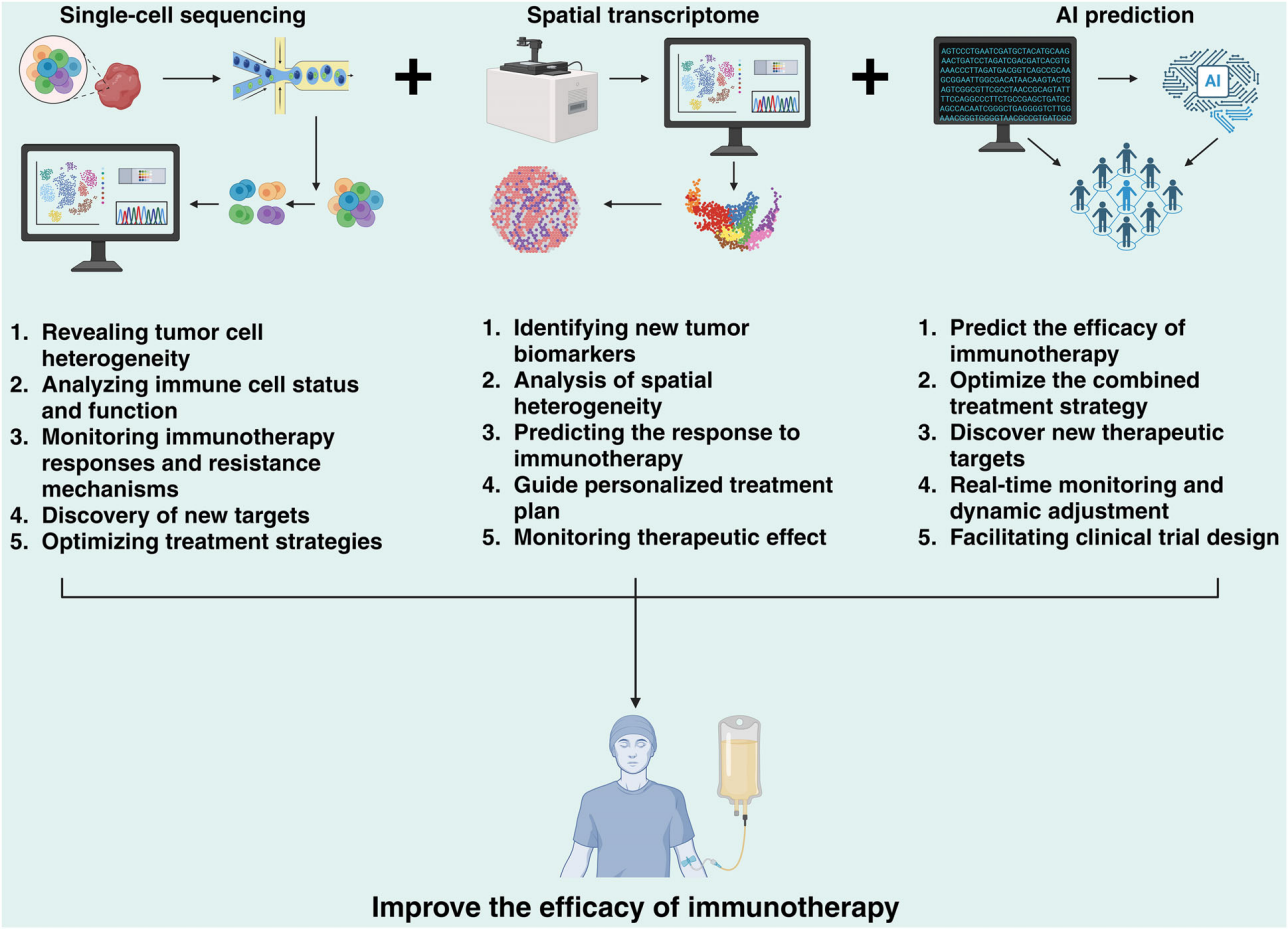
Multimodal technologies optimize anti‐tumor immunotherapy through integrated single‐cell, spatial, and AI frameworks. This figure illustrates how the integration of single‐cell sequencing, spatial transcriptomics, and AI‐based prediction transforms complex patient data into actionable insights to enhance immunotherapy efficacy. Single‐cell sequencing provides a high‐resolution dissection of tumor and immune cell heterogeneity, enabling patient stratification, novel biomarker discovery, and mechanistic insights into therapy response and resistance. Spatial transcriptomics complements this by mapping the spatial organization and interactions within the tumor microenvironment, linking immune phenotypes to their tissue context and revealing the origins of localized immune activation or suppression. AI prediction modules integrate these multimodal features to forecast therapeutic outcomes, guide personalized and combination treatment strategies, and support real‐time monitoring. Collectively, these outputs serve as practical tools to: (i) monitor treatment responses and resistance mechanisms, (ii) discover new therapeutic targets, and (iii) optimize strategies by identifying rate‐limiting immune‐regulatory bottlenecks (e.g., in immune composition, spatial architecture, or clinical predictors). This integrated approach strengthens decision‐making from prognosis stratification to the design of mechanism‐guided combination therapies.

The integration of single‐cell sequencing, spatial transcriptomics and AI is transforming precision immunotherapy, providing high‐resolution insights. Its value has been demonstrated in patient stratification, biomarker discovery and treatment optimization (Figure 5). Having established that checkpoints, immunomodulators, resistance mechanisms, and regulated cell death collectively form an interconnected immune regulatory network, a critical question remains: how can we identify, in individual patients, which network nodes are rate‐limiting at any given time? Multimodal technologies now provide the resolution needed to translate this network framework into actionable patient stratification and therapeutic optimization.

In patient prognosis stratification and treatment decision‐making, single‐cell sequencing provides a new dimension for prognostic assessment by resolving cellular composition heterogeneity within the tumor immune microenvironment. For example, in non‐small cell lung cancer, single‐cell data can classify patients into distinct immune microenvironment subtypes. Subtypes enriched with fibroblast growth factor binding protein 2 (FGFBP2)^+^ NK cells, memory B cells, and effector T cells demonstrate superior response to PDCD1 inhibitors, while those dominated by CCR8^+^ Treg cells carry the poorest prognosis. This provides direct evidence for clinically differentiated treatment strategies [184].

Spatial transcriptomics technology further guides stratification by revealing spatial organization within cells. In colorectal cancer, SPP1^+^ macrophages and SELENOP^+^ macrophages spatially colocalize and form an interactive network. Their combined spatial infiltration advantage determines patient survival and the efficacy of immunotherapy [185]. Artificial intelligence models such as the logistic regression–based immunotherapy response score (LORIS) can accurately predict survival outcomes for pan‐cancer patients receiving immune checkpoint inhibitor therapy by integrating six simple clinical features including tumor mutational burden (TMB), treatment history, and serum albumin. Its predictive efficacy surpasses traditional biomarkers and effectively identifies patient groups with low TMB who may still benefit from treatment, providing a powerful tool for clinical decision‐making [186]. Similarly, the SCORPIO model further simplifies predictive dimensions, enabling efficient stratification using only blood count metrics (e.g., NLR) and basic clinical data, making it particularly suitable for resource‐limited settings [187]. The multimodal transformer with unified maSKed modeling (MUSK) overcomes limitations of single‐data‐type models by integrating pathology images, genomic data, and clinical text for end‐to‐end prediction, though its training data scale and diversity remain areas for improvement [188].

These technologies can pinpoint the critical junctures of treatment failure, revealing drug resistance mechanisms and identifying actionable targets. For example, combined single‐cell and spatial multi‐omics analysis revealed that targeting glutathione s‐transferase pi 1 (GSTP1) in heme binding protein 2 (HEBP2)‐overexpressing tumor cells reduces glutamine‐consumption‐dependent CCL3^+^ macrophage ferroptosis, thereby enhancing antitumor immunity in mouse models of breast cancer [189]. Similarly, in metastatic melanoma, mesenchymal‐like (MES) cancer cells activate MES pathways via the transcription factor 4 (TCF4) while suppressing melanocyte differentiation and antigen presentation, leading to dual resistance to targeted therapies and ICBs [190]. Moreover, single‐cell spatial transcriptomics analysis revealed that neoadjuvant chemoradiotherapy reshapes the CAF‐malignant cell ligand‐receptor network within the PDAC tumor microenvironment, where activation of IL6 family signaling pathways constitutes a core mechanism of chemotherapy resistance [191]. Notably, these examples illustrate how multi‐omics can “localize” resistance to specific network layers (tumor‐intrinsic programs, myeloid/stromal circuits, or cell‐death‐linked metabolic states), thereby informing mechanistically aligned combinations rather than empiric regimen escalation.

In dynamic monitoring and endpoint biomarker development, single‐cell technology enables tracking of dynamic changes in immune cell composition during treatment. For instance, in d‐MMR/MSI‐H colorectal cancer, PDCD1 inhibitor treatment increased proportions of CD8^+^ TEMs, CD4^+^ Th cells, B cells, and HLA‐DRA^+^ endothelial cells, while reducing D8^+^ Trm‐mitotic cells, Tregs, IL1B^+^ monocytes, and CCL2^+^ fibroblasts decreased [192], providing cellular‐level molecular evidence for immunotherapy.

In summary, the synergistic integration of single‐cell sequencing, spatial transcriptomics, and AI is substantially advancing cancer immunotherapy toward greater precision and efficacy by translating fundamental research discoveries into actionable stratification strategies, target protocols, and biomarkers for clinical practice.

## 7 Conclusion and Future Perspectives

Cancer immunotherapy has revolutionized oncology, with immune checkpoint blockade and engineered cell therapies demonstrating that durable tumor control can be achieved through immune modulation. These successes have also revealed a fundamental principle: tumor–immune interactions are governed by context‐dependent regulatory networks rather than a single dominant axis. Accordingly, the focus of future research should shift from discovering new checkpoints toward understanding when, where, and in whom specific regulatory pathways become rate‐limiting. Intrinsic tumor programs, the tumor microenvironment (TME), systemic physiology, the microbiome, and neuroimmune signaling jointly shape therapeutic dependence and resistance.

Within this framework, the components discussed throughout this review—immune checkpoints (including metabolic checkpoints), immunomodulators, resistance mechanisms, regulated cell death programs, and multimodal technologies—should be viewed as interdependent layers of a unified tumor immune regulatory network. Mapping the dominant bottlenecks across these layers in a given tumor context is essential for explaining heterogeneous clinical responses and for designing rational, biomarker‐guided combination strategies that improve durability while managing toxicity.

Next‐generation technologies—single‐cell and spatial profiling coupled with integrative modeling—are enabling higher‐resolution views of these circuits, including cell–cell interactions, spatial constraints, and therapy‐driven dynamics. A key challenge, however, is to move beyond descriptive atlases. Priorities should include: (i) linking multi‐omics maps to perturbation‐based validation in models that preserve clinically relevant immune–stromal architecture and metabolic competition; and (ii) shifting biomarker development from static baselines toward dynamic, mechanism‐linked readouts that capture on‐treatment state transitions underlying adaptation and resistance. Identifying potential bottlenecks through spatial and single‐cell atlases, followed by genetic or pharmacologic validation, and establishing longitudinal sampling frameworks to track immune state trajectories during treatment will further enhance immunotherapy efficacy.

Despite clinical progress, persistent barriers remain. First, resistance arises from an immunosuppressive TME shaped by tumor plasticity and extrinsic cues, further complicated by systemic influences such as the gut microbiota and neuroimmune regulation. Rather than treating these as confounders, future work should define causal links and boundary conditions—which microbial functions or neural states reset immune set points, and under which therapeutic contexts they matter. Second, immune‐related toxicities limit treatment persistence and quality of life, while predictive biomarkers remain largely confined to PD‐L1 expression and tumor mutational burden. A pragmatic direction is to bias immune activation toward tumor‐restricted compartments while developing monitoring frameworks that detect early signs of dysregulation. Third, gaps between preclinical models and human biology persist, particularly for multi‐organ regulation involving neural signaling and microbial ecology; more physiologically relevant systems that integrate immune, stromal, and spatial features are urgently needed. Fourth, combination therapies are effective but mechanistically heterogeneous, and outcomes can be influenced by distinct regulated cell death patterns [193]. Future combinations should be mechanism‐guided, selected and sequenced based on measurable bottlenecks and evolving immune states.

Finally, regulated cell death should be considered not merely a cytotoxic endpoint, but a tunable immunological signal that shapes antigen availability, inflammatory tone, and immune recruitment. Its relevance to immunotherapy lies in its capacity to influence whether checkpoint blockade encounters an immune‐permissive or immune‐restrictive tumor ecosystem [150]. The central challenge is controllability, because immunogenic benefit and tissue damage may be closely linked. Future strategies should therefore prioritize tumor‐selective, spatially confined, and temporally controlled induction of specific death programs, while clarifying which modalities can synergize with ICIs without increasing systemic toxicity.

Overall, the next generation of precision immunotherapy will require a unified mechanistic view that connects checkpoint signaling, resistance networks, regulated cell death, and therapeutic optimization. Progress will depend on more physiologically relevant experimental models and closer integration among immunology, oncology, systems biology, and computational science. Such efforts should improve patient stratification, enable mechanism‐guided combinations, and ultimately support durable and safe immune‐mediated tumor control.

## Author Contributions

All authors wrote, revised, and approved the manuscript.

## Conflicts of Interest

The authors declare no conflict of interest.

## Data Availabilty Statement

The authors have nothing to report.
